# Band Alignment in Ultrathin Mixed Conducting Oxide Layers

**DOI:** 10.1021/acsami.6c08854

**Published:** 2026-06-10

**Authors:** Claudia Steinbach, Alexander Schmid, Markus Kubicek, Andreas Steiger-Thirsfeld, Michael Stöger-Pollach, Alexander K. Opitz, Jürgen Fleig

**Affiliations:** † 27259 TU Wien, Institute of Chemical Technologies and Analytics, Vienna 1060, Austria; ‡ Christian Doppler Laboratory for Oxygen-Ion Batteries, Vienna 1060, Austria; § TU Wien, University Service Centre for Transmission Electron Microscopy, Vienna 1040, Austria

**Keywords:** space charges, SrTiO_3_, mixed ionic electronic conductors, electrochemical impedance spectroscopy, interlayer, thin films

## Abstract

At heterojunctions between mixed ionic and electronic conductors (MIECs), band alignment takes place in order to equilibrate electronic and ionic charge carriers. The bulk properties of such MIECs can be used to describe and understand the corresponding interfacial space charges. For ultrathin films in MIEC heterolayers, however, these interfacial effects may differ from those between bulk materials. In this work, the interfacial regions between SrTiO_3_ (STO) and the two MIECs (La, Sr)­FeO_3−δ_ (LSF) and (La, Sr)­MnO_3−δ_ (LSM) are considered. Stacks of LSF|LSM and LSM|LSF were deposited on STO single crystals, and the resulting space-charge regions in STO are characterized by means of impedance spectroscopy at 500 °C in the *p*(O_2_) range between 1 and 5 × 10^–4^ bar. By extracting the STO bulk and space-charge resistances from the impedance data, space-charge potentials are derived. Interestingly, even extremely thin LSM interlayers (0.5 nm) cause bulk-like LSM band bending beneath LSF top layers. STO space charges at LSF interlayers of the same thickness, however, are strongly affected by LSM top layers. A model is introduced to interpret the measured difference in critical thicknesses of LSF and LSM in terms of different accumulation- and depletion-layer thicknesses in MIECs.

## Graphical abstract

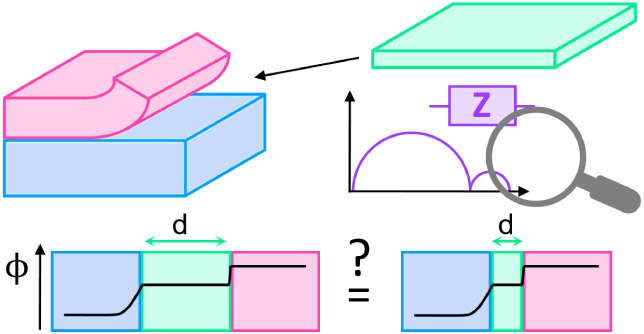

## Introduction

Mixed ionic and electronic conductors (MIECs) are a class of materials that exhibit both ionic and electronic conductivity. This mixed conduction enables the use of MIECs in applications where both ion and electron transport is necessary or at least helpful, such as in fuel cells, batteries and supercapacitors.^1–7^ Oxides displaying comparatively high electronic conductivity, such as (La, Sr)­FeO_3_ (LSF) or (La, Sr)­CoO_3_ (LSC), are often used as cathode materials in fuel cells, where the mixed conductivity is highly beneficial for the electrochemical reaction of the fuel cell.^8–11^ Large band gap perovskites, such as SrTiO_3_ (STO) or BaTiO_3_ (BTO), form another group of MIECs, where the ionic and electronic conducting properties can be deliberately tailored from insulating to conducting.^12,13^ This makes STO or BTO widely applicable in sensors or capacitors.^14–17^

The mixed conductivity in such MIECs is heavily influenced by their defect chemistry and the corresponding thermodynamic data, such as electronic band gap or oxygen vacancy formation energy, as those dictate the relative contribution of ions and electrons to the conductivity in dependence of the experimental conditions (e.g., temperature or oxygen partial pressure). As a result, there are many studies focusing on the bulk properties of mixed conducting oxides, and defect chemical models exist to describe the ionic and electronic bulk defect concentrations.^18–21^ Also many studies on oxygen surface exchange kinetics are found in literature,^22–26^ often focusing on bulk-like materials and more recently also on the effects of surface decorations on the oxygen incorporation reaction.^27,28^

Interfaces between MIEC heterojunctions are far less investigated and models describing those interfacial effects are rarely found. Particularly interesting in this context is the fact that not only electronic charge carriers have to equilibrate by band alignment but also ionic defects have to equilibrate at the interfaces, while at the same time all MIECs are in a further equilibrium with oxygen in the gas phase.^6,29^ Moreover, ionic and electronic defect concentrations are interwoven by the corresponding defect thermodynamics, which might even depend on the geometry when turning to layers of only few nm thickness. Studies showed, for example, that properties of nanoparticles or thin films might differ from their bulk counterparts.^18,23,30,31^

A previous work^29^ focused on investigating the space charges at different STO|MIEC interfaces resulting from STO single crystals and LSF, LSC, LSM, LSCr ((La, Sr)­CrO_3_) and YBCO (YBa_2_Cu_3_O_7_) thin films of approximately 50 nm thickness. A model was introduced to describe and predict space charge potentials based on ionic and electronic defect species, their interplay in the space charge region and defect thermodynamic data. This model defines the general framework of how space charges at MIEC heterojunctions are formed and depend on the defect chemistry of the used materials. However, in that study the films were sufficiently thick that only little deviation from bulk behavior was expected. The question remained, whether or not ultrathin (inter)­layers on STO single crystals still lead to the same band bending. This question is also of relevance in view of the effects of surface decorations on interfacial properties.

In order to resolve this question, the given work reports on impedance measurements of heterojunctions between nominally undoped STO single crystals and thin film layer stacks consisting of (primarily) hole conducting LSF (La_0.6_Sr_0.4_FeO_3−δ_) and LSM (La_0.65_Sr_0.35_MnO_3−δ_) at 500 °C in high oxygen partial pressures (1 to 5 × 10^–4^ bar). The interlayer thicknesses are varied between (nominally) 0.1 and 10 nm. From impedance data, space charge potentials are derived to characterize the space charge regions in the STO|LSF|LSM and STO|LSM|LSF samples and their dependence on the interlayer thicknesses (LSF and LSM, respectively). The previously developed model is used to interpret the film thickness dependence of the space charge potential and thus the main conclusions are expected to be applicable also to other MIEC heterojunctions.

## Experimental Section

### Sample Preparation

The samples used for impedance measurements are based on nominally undoped STO (100) single crystals (10 × 10 × 0.5 mm^3^, Crystec (Germany), both sides polished), which served as substrates after cleaning with ethanol. Pulsed laser deposition (PLD) was used to deposit MIEC thin films onto the STO substrates. First, an approximately 50 nm thick YBa_2_Cu_3_O_7−δ_ (YBCO) layer was deposited onto the backside of the STO single crystals to serve as a reversible (ohmic) counter electrode, as YBCO does not show a measurable space charge on STO.^29,32^ On the top side of the STO single crystal, a thin interlayer (between 0.5 and 10 nm) of either LSF or LSM was deposited. Even a sample with nominally 0.1 nm LSM (i.e., partial monolayer coverage) was prepared. An approximately 50 nm top layer of either LSM (on LSF) or LSF (on LSM), respectively, was then deposited from a second target directly on top of the thin interlayer, without breaking vacuum/deposition conditions. For comparison, approximately 50 nm solo thin films (without interlayer) were also deposited onto STO single crystals with YBCO counter electrodes. The used PLD parameters are listed in Table 1. Film thicknesses were determined by the use of a quartz crystal microbalance (QCM). Each film was deposited at a target-substrate distance of 6 cm and a pulse frequency of 5 Hz. At these conditions, films were grown at 60 laser shots per nm or ca. 0.08 nm/s.

**Table 1:** PLD Parameters for the MIEC Thin-Film Depositions^a^

MIEC thin film	*p*(O_2_)	Temperature	Fluence
YBa_2_Cu_3_O_7−δ_ (YBCO)	3 × 10^–1^ mbar	800 °C	1.5 J cm^–2^
La_0.6_Sr_0.4_FeO_3−δ_ (LSF)	4 × 10^–2^ mbar	600 °C	1 J cm^–2^
La_0.65_Sr_0.35_MnO_3−δ_ (LSM)	4 × 10^–2^ mbar	600 °C	1 J cm^–2^

a The distance between the target and the substrate was set to 6 cm and the pulse frequency to 5 Hz.

On both the bottom and top side, Pt current collector grids were applied by means of photolithography and subsequent DC-magnetron sputtering (Baltec MED020). A 5 nm layer of Ti (grid) was deposited between the MIEC films and the Pt grid and acted as adhesion layer for a 100 nm Pt layer. Grids with 15 μm strip width and 35 μm mesh distance were used. The used sample geometry is sketched in Figure 1.

**Figure 1:**
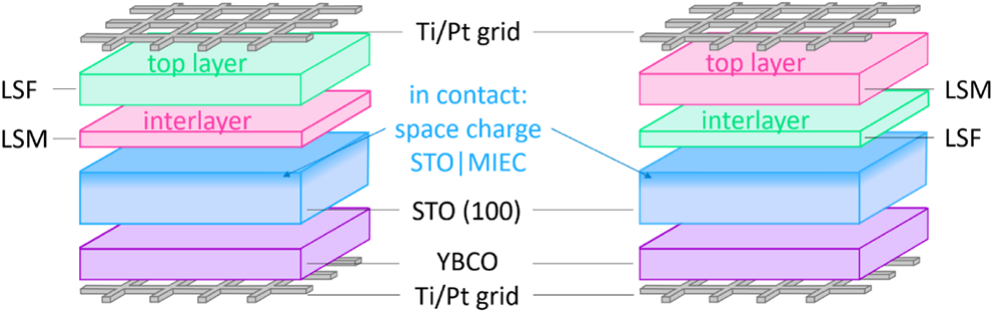
Schematic illustration of the used interlayer sample geometries for the LSM|LSF|STO and LSF|LSM|STO stacks. The shown thicknesses are not to scale.

A detailed analysis of the interfacial regions of the investigated heterolayer-stacks was performed using TEM. For this purpose a FEI TECNAI field emission TEM equipped with a super twin objective lens was used, operated at 200 keV beam energy. The TEM is equipped with a GATAN GIF Tridiem energy filter and an EDAX-AMETEK Apollo XL 12 energy dispersive X-ray spectrometer. It can be operated in TEM mode as well as in scanning TEM (STEM) mode for simultaneously recording images and EELS and EDX spectra with high spatial resolution.

### Measurement Setup and Electrochemical Characterization

Impedance measurements were performed using a Novocontrol Technologies Alpha-A High Performance Frequency Analyzer and a 4 Wire Impedance Test Interface. Impedance data was recorded in a frequency range from 1 MHz to 30 mHz with an applied AC_RMS_ voltage of 20 mV. The temperature was set to 500 °C throughout the measurements. The oxygen partial pressure *p*(O_2_) was varied between 1 bar and 5 × 10^–4^ bar by mixing O_2_ and N_2_ gas. The total pressure was held at atmospheric conditions by a pressure relief valve. Initially and after each *p*(O_2_) change the resistance of each sample was tracked over time and a change of less than 1.5% of the resistance between four consecutive impedance measurements was used as an indication of sufficient equilibration with the corresponding gas phase. Thus, also potentially existing oxygen nonequilibria established during preparation are no longer relevant. Typical equilibration times were ≥24 h. The impedance spectra taken in the equilibrium state were fitted using the software ZView (Scribner). The fit parameters were then used to calculate further parameters, such as the space charge potential.

## Results and Discussion

### Sample Characterization

A thorough analysis of nominally identical solo LSF thin films grown with these parameters, including an X-ray diffractogram, atomic force microscopy (AFM) and transmission electron microscopy (TEM) images, as well as a diffractogram of nominally identical LSM, also grown with these parameters, is published elsewhere.^29^ There, it was shown that nominally identical LSF and LSM thin films grow epitaxially on STO (100) single crystals.

An AFM image taken of an LSM interlayer without top layer is given in Figure 2. The root-mean-square roughness of the approximately 4 nm thin film amounts to 157 pm, indicating a very smooth surface. Moreover, terraces stemming from the STO (100) single crystal beneath the LSM thin film are still detectable, suggesting (in combination with the diffractograms of other identically prepared thin films) epitaxial growth of the LSM (and also LSF) on STO. The very low roughness also suggests that even nominally 0.5 nm thick films (ca. 1.2 times the lattice parameter) are dense and that the top layers have little, if any, direct contact with STO.

**Figure 2:**
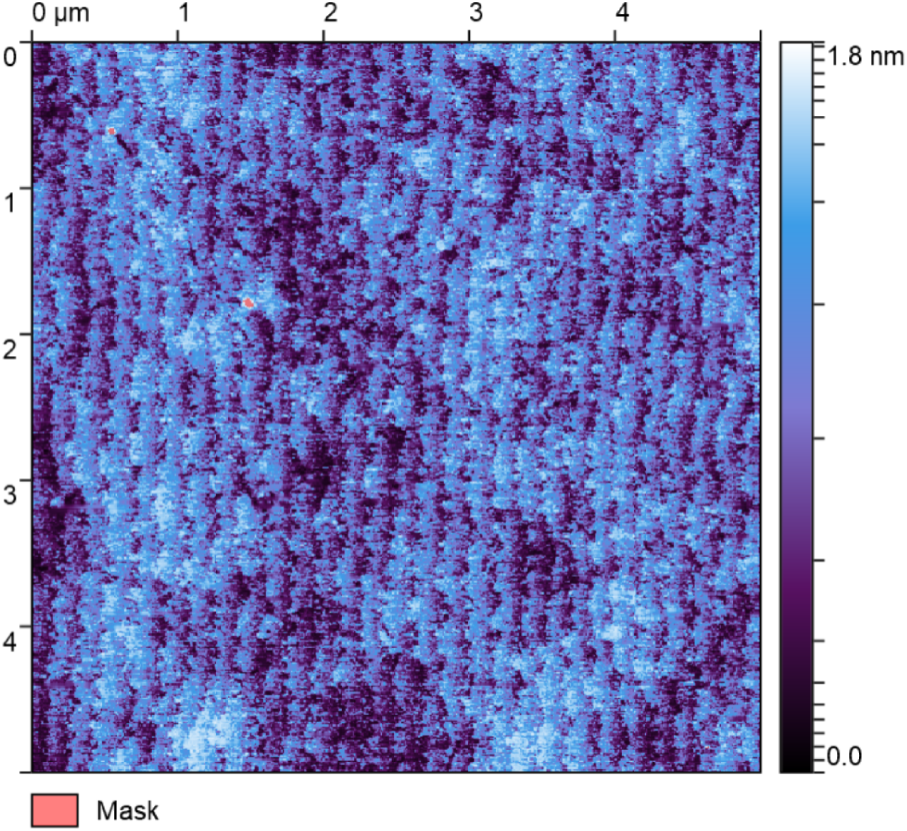
AFM image taken of a 4 nm LSM thin film grown on STO (100).

A detailed interfacial analysis of the investigated heterolayer-stacks was performed using TEM. Figure 3 presents electron microscopy images taken of an LSM|LSF|STO heterolayer. According to quartz microbalance measurements inside the deposition chamber, the LSF interlayer thickness is expected to be 2.5 nm. Figure 3a shows a high resolution TEM (HRTEM) image of an LSM|LSF|STO heterolayer, where the LSF interlayer thickness amounts to 2.7 nm. A high resolution scanning TEM (HRSTEM) image of the same heterolayer is presented in Figure 3b, showing an atomically flat interface between the STO single crystal and the dense MIEC heterolayers on top. A further STEM image is given in Figure 3c, with the scanning direction being indicated with the light purple arrow. The measured film thickness of the LSF interlayer is 2.6 nm. Electron energy loss spectroscopy (EELS) measurements were conducted in parallel to STEM and the results are given in Figure 3d. The Fe signal is almost exclusively found in a very thin layer at the very interface of the STO single crystal, i.e., where the LSF layer is supposed to be. Figure 3e shows the Gaussian fit of the Fe signal, measured by EELS. The full width at half-maximum (fwhm) amounts to 2.48 nm. Overall, the measured film thicknesses via electron microscopy are comparable with the film thicknesses determined by the quartz microbalance before PLD deposition of the thin films. These and further electron microscopy measurements (provided in the Supporting Information, chapter S1) suggest very sharp interfaces with little cation intermixing. This is also in accordance with literature, where very sharp interfaces were reported between STO and LSM or STO and other mixed conductors, such as SrRuO_3_ or Ca_0.5_Sr_0.5_IrO_3_, and enable, for instance, fabrication of superlattices.^33–35^

**Figure 3:**
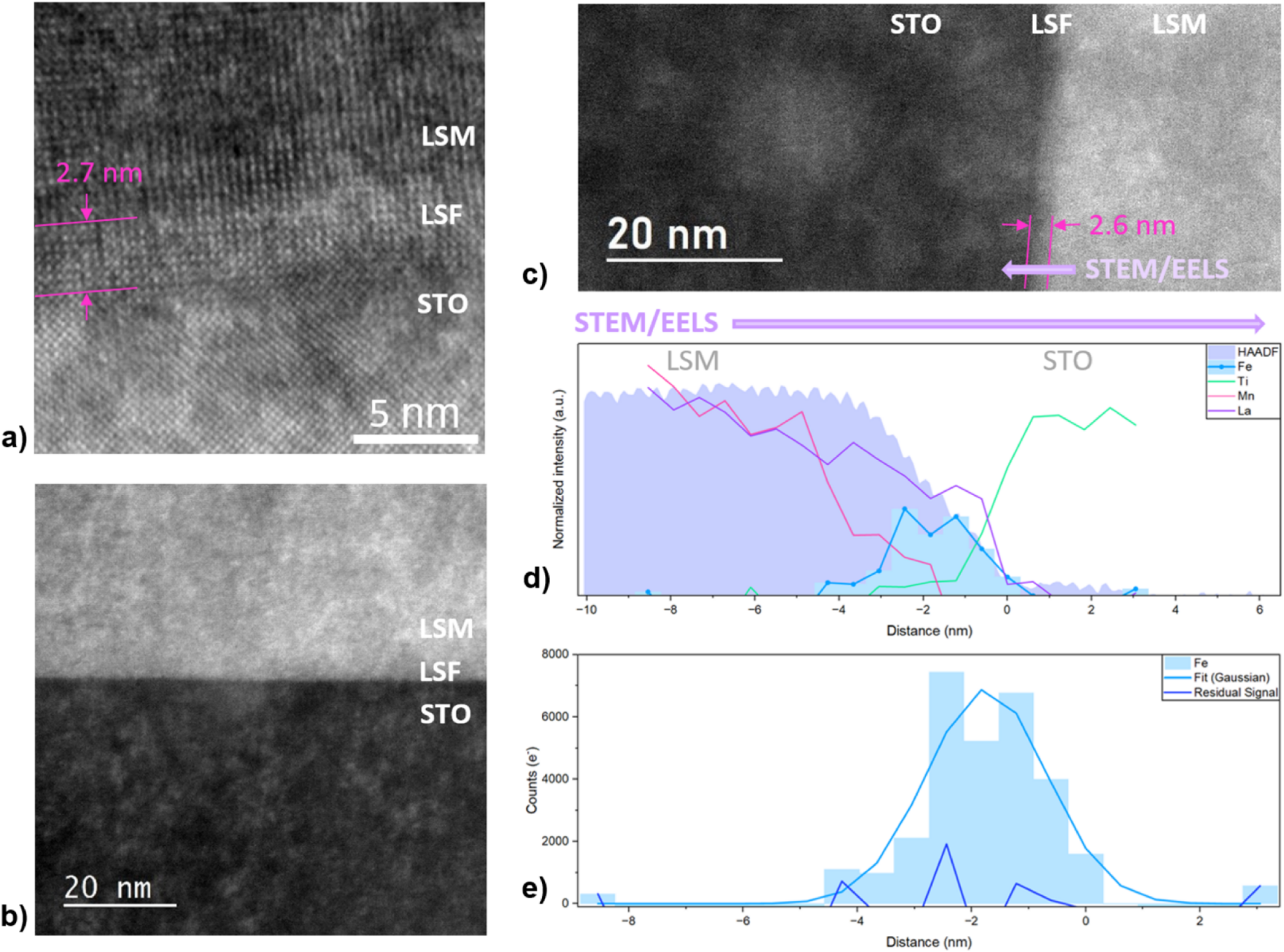
(a) HRTEM image showing an LSF interlayer of 2.7 nm between the STO single crystal and the LSM top layer. b) HRSTEM image of the same heterolayer showing an atomically flat interface between the STO single crystal and the mixed conductors on top. c) STEM image of the same interface, showing an LSF layer growth of 2.6 nm. The light purple arrow indicated the scanning direction. d) EELS measurement parallel to STEM indicating that Fe can be found at the very interface of the sample. e) Gaussian fit of the EELS measured Fe signal yielding a fwhm (full width at half-maximum) of 2.48 nm.

### Impedance Spectra and Their Analysis

Nyquist plots for the samples with LSF interlayers and LSM top layers are exemplarily shown for some thicknesses in Figure 4, all measured at 200 mbar *p*(O_2_) and 500 °C. For comparison, spectra of solo LSF and LSM thin films are plotted as well. The impedance data show a high frequency arc of very similar size for all samples. It is assigned to the STO single crystal bulk and arises from the mixed ionic and electronic conductivity in STO. For the considered *p*(O_2_) range and temperature, primarily hole conduction can be expected in our nominally undoped STO substrates.^19^ Indeed, the corresponding bulk conductivity is in excellent agreement with hole conductivity values and partial pressure dependencies measured for such nominally undoped STO single crystals.^19^ The capacitance of this high frequency arc is around 2.8 × 10^–10^ F/cm^2^ for all samples at 200 mbar *p*(O_2_), corresponding to the geometrical capacitance of the STO single crystal and a relative permittivity of 150 to 160, which are typical values reported for STO.^36,37^

**Figure 4:**
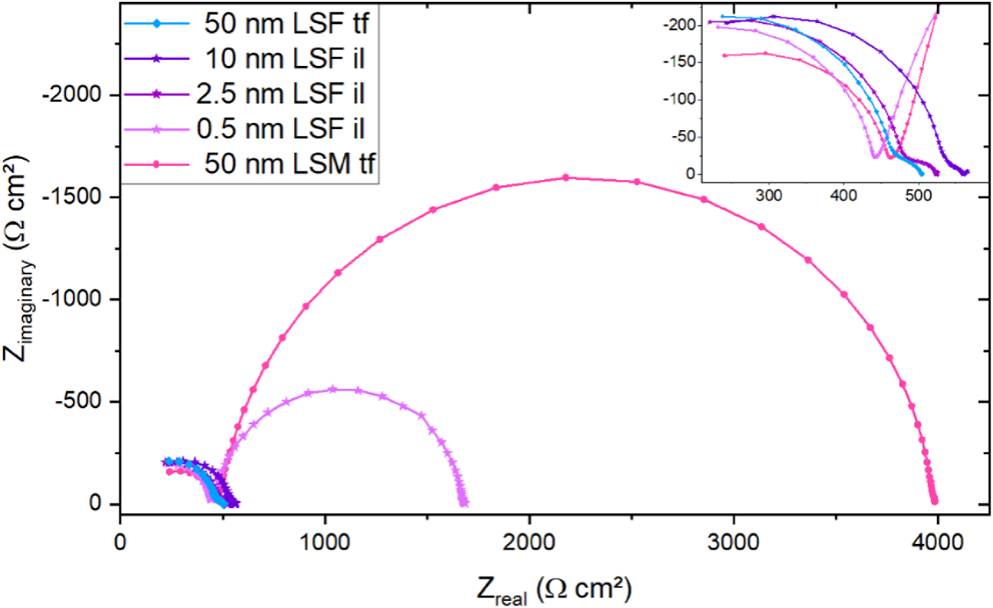
Impedance plots for the LSF interlayer samples (il), as well as reference impedance spectra for solo LSF and LSM thin films (tf) at 500 °C and 200 mbar *p*(O_2_).

At intermediate to low frequencies, impedance data show a second semicircle, whose magnitude depends on the combination of MIEC and interlayer on top of STO. In agreement with previous studies,^29,38,39^ this mid frequency arc or shoulder is attributed to the space charge of the MIEC|STO interface, as the capacitance values (around 6 × 10^–7^ F/cm^2^ for LSF thin films and around 1 × 10^–6^ F/cm^2^ for LSM thin films) are typical values expected for such space charges in undoped STO. Its resistance reflects the depletion of holes in STO due to band alignment between STO and the MIEC by space charge formation. Furthermore, also for the investigated MIECs (LSF and LSM) primarily hole conduction can be expected in the parameter range of this study.^18,21^ As a consequence, additional ionic resistances, including oxygen surface exchange resistances, are irrelevant and should not cause any additional impedance features. Also impedance contributions from any space charges are absent for our YBCO|STO interfaces as shown in reference 29. Further details on the interpretation of the spectra are given in an extensive study on solo layers.^29^

The smallest space charge feature is found for the solo LSF thin film and the thickest LSF interlayer of 10 nm. For thinner LSF interlayers the space charge contribution increases, though for 2.5 nm the space charge arc is still only slightly larger than for the 10 nm interlayer and the solo LSF thin film. However, 0.5 nm LSF interlayers lead to a rather prominent space charge semicircle. The largest space charge feature was measured for a solo LSM thin film.

Figure 5 shows the measured impedance data for some LSM interlayers with LSF top layers, recorded at 200 mbar and 500 °C, again together with impedance spectra for LSF and LSM solo thin films on STO. Similar to thicker LSF interlayers, thick LSM interlayers (4 nm) show a space charge feature that is of similar size as the space charge arc of the solo LSM thin film. Remarkably, already an interlayer of nominally 0.1 nm LSM, which is nominally only 25% of a unit cell and thus rather a kind of inhomogeneous surface decoration of STO, leads to a space charge arc that is about a factor of 3.5 larger than the space charge feature for the solo LSF thin film. Additional impedance data, showing the *p*(O_2_) dependence exemplarily for the 2.5 nm LSF and the 2 nm LSM interlayers are given in the Supporting Information, S2.

**Figure 5:**
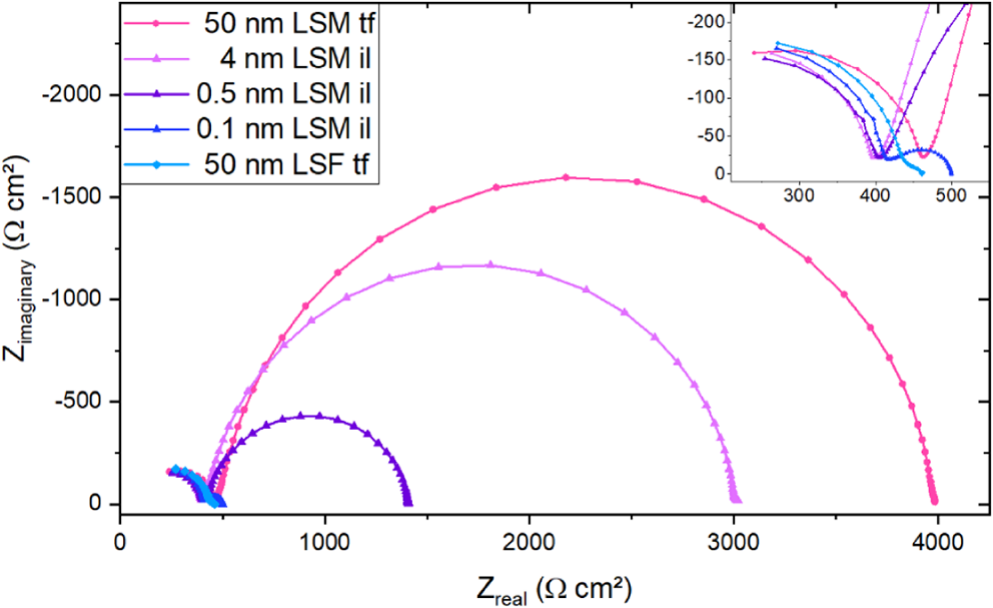
Impedance plots for the LSM interlayer samples (il), as well as reference impedance spectra for solo LSF and LSM thin films (tf) at 500 °C and 200 mbar *p*(O_2_).

Impedance data were fitted using the nested equivalent circuit shown in Figure 6a. This circuit was chosen over two serial RC-elements, as especially for small space charge features it takes better into account that both the capacitive counter charge of the STO space charge and the charge of the geometrical sample capacitor are located in one and the same MIEC layer. *R*_STO_ is the mixed ionic and electronic resistance of the STO bulk, with the main contribution being from electronic (holes). *C*_STO_ is the dielectric capacitance of the STO single crystal. *R*_SC_ and *C*_SC_ are the space charge resistance and capacitance of the LSM|LSF|STO and LSF|LSM|STO heterostructures, respectively. The space charge capacitance was fitted using a constant phase element (CPE) to improve the fit quality. The impedance of a CPE element is $Z_{\mathrm{CPE}}=\frac{1}{{\left(j\omega \right)}^{\alpha }Q}$ and the corresponding capacitance is calculated through $C_{\mathrm{CPE}}={\left(R^{1-\alpha }\times Q\right)}^{1/\alpha }$ . As detailed below, the band alignment takes place largely in the very thick space charge of the nominally undoped STO and thus *R*_SC_ reflects primarily the resistance of the hole depletion layer in STO. The inductive element is implemented to take account of any inductive effects caused by the measurement wires and cables within the measurement configuration.

**Figure 6:**
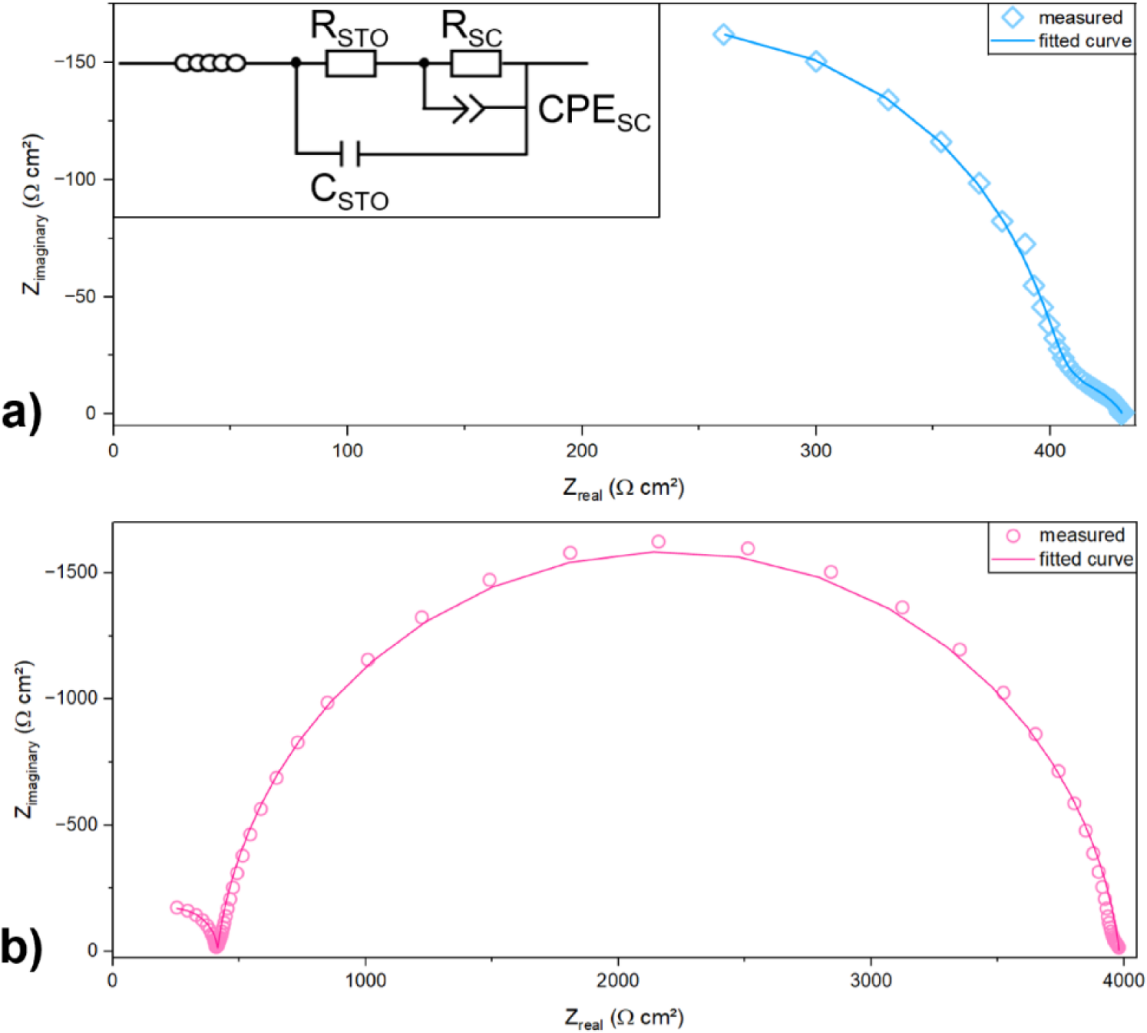
(a) Equivalent circuit used to fit measured impedance data. *R*_STO_ and *R*_SC_ are the STO bulk and the space charge resistance, respectively. *C*_STO_ and *CPE*_SC_ are the STO capacitance and the constant phase element used to fit the capacitive contribution of the space charge, respectively. The inductive element is implemented to counteract any inductive effects caused by the parallel routing of measurement wires and cables within the measurement configuration. Measured impedance data is shown for a 50 nm LSF solo thin film on top of STO (100) at 200 mbar and 500 °C. The fitted curve was obtained by using the presented equivalent circuit. b) Measured impedance data of a 50 nm LSM solo thin film on top of STO (100) at 200 mbar and 500 °C. The fitted curve was also obtained using the presented equivalent circuit.

Figure 6a shows exemplarily the measured and fitted impedance data for a 50 nm LSF solo thin film on top of STO at 200 mbar and 500 °C, which exhibits the smallest space charge feature of the investigated samples. Typical fitting errors for *R*_STO_ and the corresponding capacitance *C*_STO_ are <1%. Fitting errors for *R*_SC_ are <2% for higher oxygen partial pressures and increase to <5% at the lowest measured *p*(O_2_) (0.5 mbar, where the space charge feature is only visible as a shoulder of the much larger STO single crystal bulk feature. Fitting errors for *C*_SC_ are around 9% at 1000 mbar and increase up to 32% at 0.5 mbar. While fitting errors for all investigated samples are the same for *R*_STO_ and *C*_STO_, an increasing space charge feature leads to a decrease in fitting errors. The largest space charge feature was found for a 50 nm LSM solo thin film on top of STO. Figure 6b shows the measured and fitted impedance data of such an LSM solo thin film. Here, the fitting errors for *R*_SC_ are <1% and around 5% for *C*_SC_ (for all *p*(O_2_)).

The fitted resistance values for the LSF interlayers are given in Figure 7. Interlayer samples are labeled il and the thickness of the interlayer is indicated. Resistances are normalized to the electrode area. The solo thin film values used for reference are labeled tf. The resistances of the STO single crystals were averaged over all samples and the error bars indicate the standard deviation. The averaged *R*_STO_ yields 378 Ω cm^2^ ± 38 Ω cm^2^ at 1 bar. It exhibits a *p*(O_2_) dependence with a slope of $-\frac{1}{4.5}$ . This is very close to the slope expected from the defect model $\left(-\frac{1}{4}\right)$ and thus supports our assumption that all samples are indeed equilibrated with the respective gas phase. (Additional long-term impedance measurements showing that the change in resistance is truly a consequence of a change in oxygen partial pressure is given in the Supporting Information, S2.)

**Figure 7:**
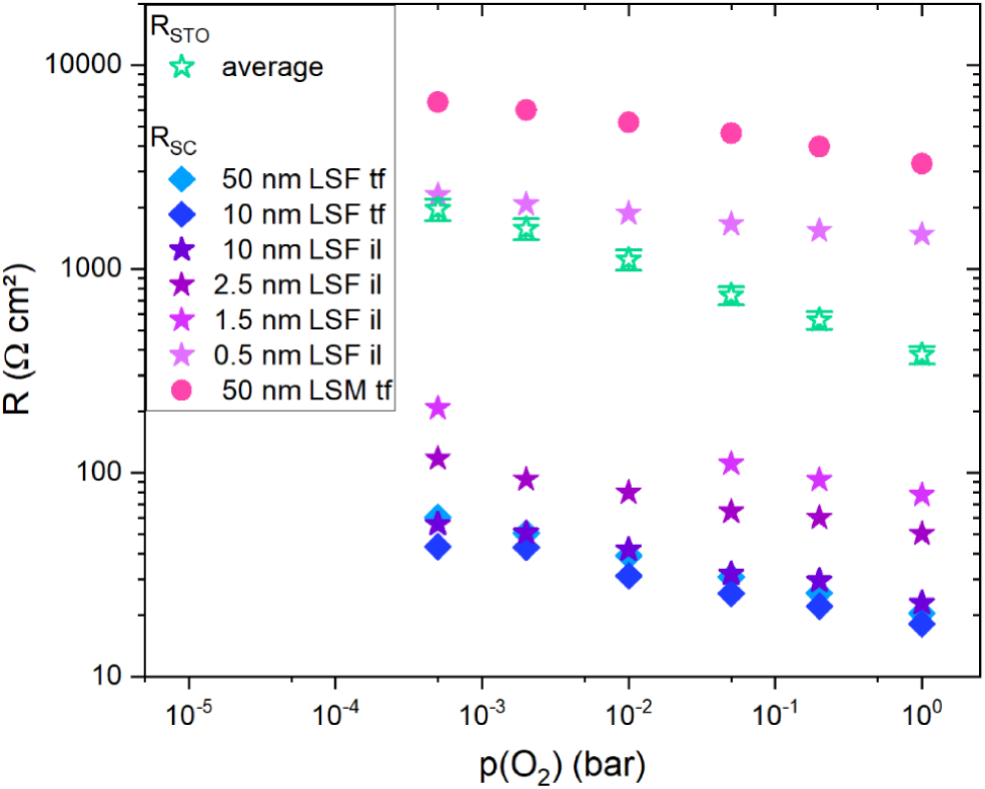
Resistance of the STO bulk *R*_STO_ and the resistance of the space charge *R*_SC_ of the interlayer (il) sample STO|LSF|LSM. The interlayer thickness is given in nm. Comparison values for *R*_SC_ of solo LSF and LSM thin films on STO (tf) are given as well.

The space charge resistances show the trends already seen for the impedance plots: The smallest space charge resistance is measured for solo LSF thin films, amounting to 20 Ω cm^2^ at 1 bar. The 10 nm LSF interlayer sample exhibits a space charge resistance that is almost identical to the solo LSF thin films. The slight deviation could be caused by scattering between the LSF layers. For thinner interlayers (≤2.5 nm) the space charge resistance increases, with the main resistance jump taking place at a critical thickness of about 1.5 nm.

The fitted resistances for the LSM interlayers are shown in Figure 8. *R*_STO_ is averaged for all samples, yielding 330 Ω cm^2^ ± 42 Ω cm^2^ at 1 bar, and the error bars indicate the standard deviation. The largest space charge resistance is found for the 50 nm solo LSM thin film and the 10 nm LSM interlayer. With decreasing LSM interlayer thickness the space charge resistance decreases. However, the main resistance jump takes place below 0.5 nm and even an LSM interlayer of nominally 0.1 nm exhibits a space charge resistance that is distinctly larger than the space charge resistance of the solo LSF thin film. The exact impact of the interlayer thickness becomes even more apparent when considering the space charge potential (Δϕ).

**Figure 8:**
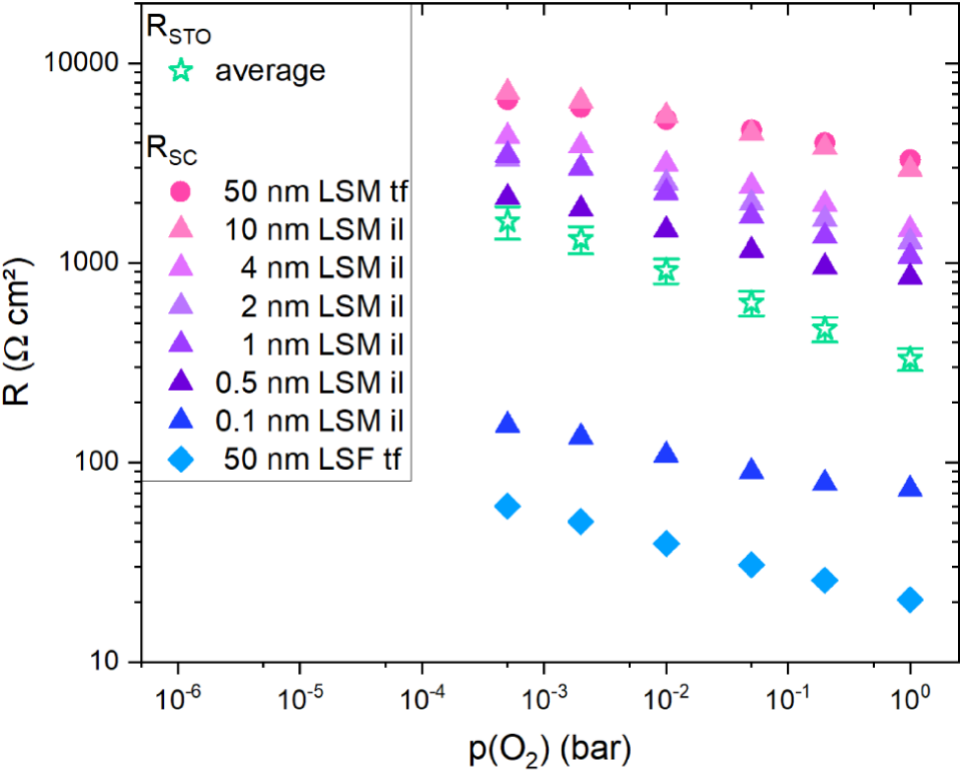
Resistance of the STO bulk *R*_STO_ and the resistance of the space charge *R*_SC_ of the interlayer (il) sample STO|LSM|LSF. The interlayer thickness is given in nm. Comparison values for *R*_SC_ of solo LSF and LSM thin films on STO (tf) are given as well.

### The Space Charge Potential

In principle, the space charge region at the interface between STO and an MIEC layer or interlayer has two contributions: one from STO and one from the contacting MIEC. However, due to the high electronic charge carrier concentrations arising from the high acceptor doping of LSF and LSM, the space charge regions in those MIECs are very thin. Consequently, we assume that virtually the entire band alignment at the STO|MIEC interface takes place in STO and thus also the entire relevant space charge region is located in STO. Actually, a further band alignment takes place at the MIEC interlayer|top layer interface. However, due to the very high hole concentration and conductivity of LSF and LSM we assume that the corresponding interface does not significantly contribute to the measured resistance. *R*_SC_ from the impedance analysis thus represents the hole depletion in the space charge of STO. Assuming validity of the drift-diffusion model and using a Schottky approximation,^29,40^ the area-specific bulk resistance *R*_STO_ and the area-specific resistance of the space charge in STO *R*_SC_ can thus be used to calculate the space charge potential Δϕ in STO according to

1 $$\frac{R_{\mathrm{SC}}\frac{1}{w_{\mathrm{SC}}}}{R_{\mathrm{STO}}\frac{1}{d}}=\frac{\exp\frac{e\Delta \phi }{kT}}{\frac{2e\Delta \phi }{kT}}$$

where d is the thickness of the single crystal, e is the elementary charge, k is the Boltzmann constant and T is the temperature. In this Schottky approximation the space charge thickness w_SC_ is given by

2 $$w_{\mathrm{SC}}=\sqrt{\frac{2\varepsilon_{r}\varepsilon_{0}\left|\Delta \phi \right|}{eN_{d}}}$$

where *ε*_0_ is the vacuum permittivity, *ε*_r_ is the relative permittivity of STO and N_d_ is the dopant concentration.

For the calculations of Δϕ and w_SC_, *ε*_r_ was assumed constant throughout the entire STO single crystal, including the space charge region. For nominally identical undoped STO single crystals, a dopant concentration *N*_d_ corresponding to 24 ppm singly charged acceptors per unit cell was reported.^19^ Solving eqs 1 and 2 numerically leads to Δϕ values for the investigated space charge zones. The fitting errors of the resistances *R*_STO_ and *R*_SC_ and uncertainties in *N*_d_ and the thickness *d* translate to an estimated error of Δϕ in the few % range. The Δϕ values obtained for the LSF interlayers sandwiched between the STO single crystals and LSM thin films are shown in Figure 9. Space charge potentials determined for solo LSF and LSM thin films are given for comparison as well. The different data points for a given interlayer thickness indicate the values for different *p*(O_2_). This slight decrease of the space charge potential with decreasing *p*(O_2_) despite increasing *R*_SC_ can be explained by the even more pronounced increase of the bulk resistance *R*_STO_ of eq 1, compare Figures 7 and 8. In ref 41 it is shown that the oxygen partial pressure dependence of Δϕ is due to the interplay of different *p*(O_2_) dependent defect concentrations in STO and MIEC. However, a detailed discussion of the oxygen partial pressure dependence of Δϕ is beyond the scope of this paper. Rather, this reproducible *p*(O_2_) dependence is taken as a clear indication that indeed equilibration with the gas phase is given.

**Figure 9:**
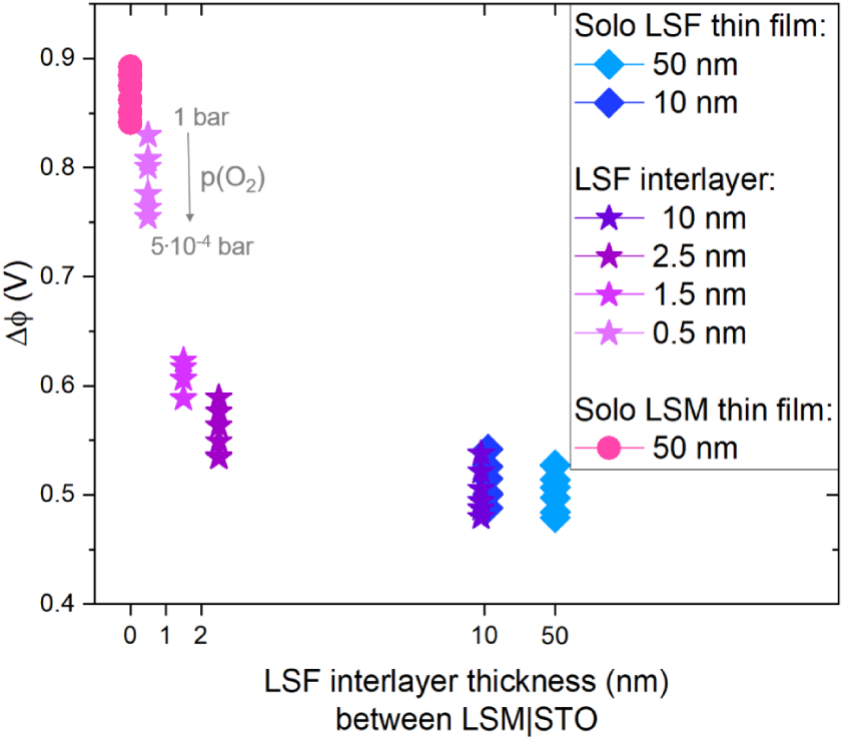
Space charge potentials in dependence of the LSF interlayer thickness for the junction STO|LSF|LSM. The different data points for a given interlayer thickness indicate the values for different *p*(O_2_).

The smallest space charge potential is found for the solo LSF thin film of 50 nm with 527 mV at 1 bar. For the 10 nm LSF interlayer, a very similar space charge potential of 542 mV was found and this difference may still be sample scattering. The 2.5 nm LSF interlayer shows a significantly larger space charge potential of 589 mV. It further increases for 1.5 nm, and for the 0.5 nm LSF interlayer it even reaches 830 mV, which is already close to the value of the solo LSM thin film. (All values at *p*(O_2_) = 1 bar.) Accordingly, either the properties of the LSF layers substantially change at 2.5 nm or the top layer LSM begins to significantly affect the space charge in STO despite the presence of approximately 1 to 2 nm LSF.

The space charge potentials for the LSM interlayers are given in Figure 10. Very similar space charge potentials are found for 50 nm solo LSM and the 10 nm interlayer sample (887 mV and 892 mV, respectively, at 1 bar). For 4, 2, and 1 nm interlayers, slight decreases of Δϕ are present but the values stay rather close to that of (bulk-like) solo LSM. Differences may reflect minor changes in LSM of different thicknesses or a very minor affect of LSF itself on Δϕ in STO. Even for an LSM interlayer as thin as 0.5 nm, the space charge potential (802 mV at 1 bar) is close to the solo LSM layer. Only for the submonolayer (0.1 nm = about 25% of a cubic ABO_3_ elementary cell) a strong decrease of Δϕ toward the solo LSF value is found.

**Figure 10:**
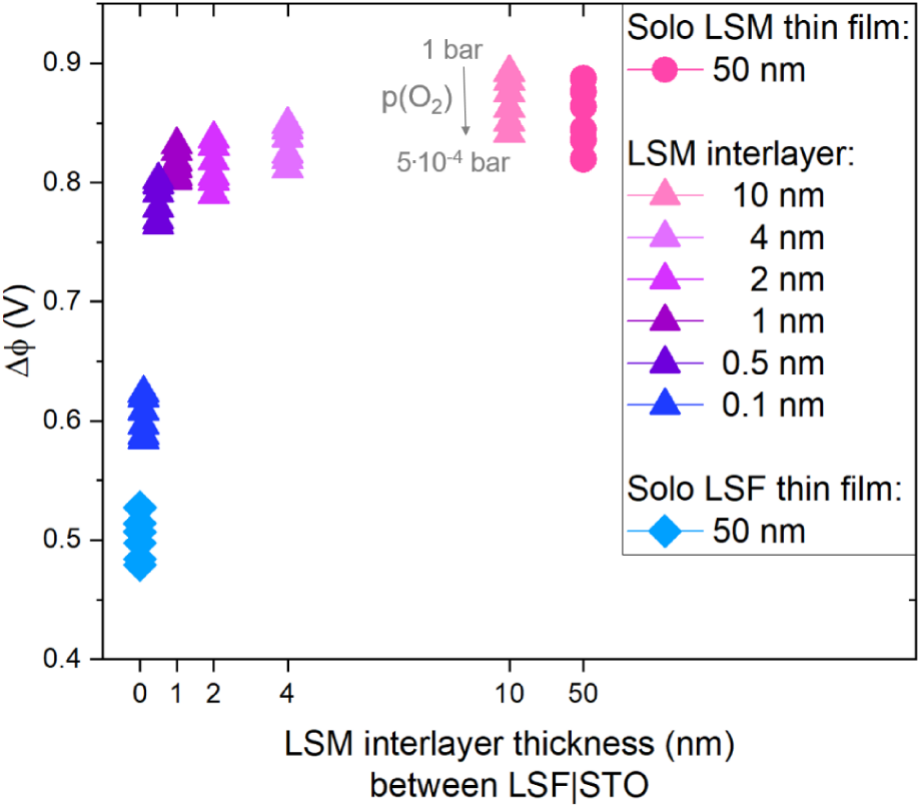
Space charge potentials in dependence of the LSM interlayer thickness for the junction STO|LSM|LSF. The different data points for a given interlayer thickness indicate the values for different *p*(O_2_).

Accordingly, the interfacial space charges and thus the band alignment for LSF and LSM interlayers show a different dependence on the film thickness. While the space charge potential of the STO|LSF|LSM stack requires >1.5 nm LSF to come close to the solo LSF value, stacks with LSM interlayers are already for 0.5 nm rather LSM bulk-like. A ″critical thickness″ of an interlayer can thus be defined as the minimal thickness where we still have (almost) bulk like properties and this critical thickness is much larger for LSF (with LSM top layer) compared to LSM (with LSF top layer). In the following considerations, we suggest a model that can explain the measured space charge potential behaviors.

### Basic Space Charge Model Considerations

The subsequent considerations are based on the model introduced in ref 29 for two mixed conducting oxides in equilibrium. The model also describes the interplay between ionic and electronic charge carriers in the space charge zone in STO and how they contribute to the space charge potential Δϕ in STO. Further, this model relates Δϕ values to the reducibilities of the contacting MIECs on top of STO. In the following, key aspects of the model, which are needed to explain the film thickness dependence of the space charge potential, are summarized.

We first consider a sample without interlayer, i.e., only STO and a solo MIEC on top. The atmospheric oxygen chemical potential $\mu_{O_{2}}^{\mathrm{at}}$ is related to the normalized oxygen partial pressure *p*(O_2_) through

3 $$\mu_{O_{2}}^{\mathrm{at}}=\mu_{O_{2}}^{0}+kT\;\ln p\left(O_{2}\right)$$

with $\mu_{O_{2}}^{0}$ being the standard chemical potential at 1 bar *p*(O_2_). If a sample with sufficiently fast oxygen exchange kinetics and an oxygen chemical potential $\mu_{O_{2}}^{\mathrm{sample}}$ is brought into this atmosphere, $\mu_{O_{2}}^{\mathrm{sample}}$ starts to align with $\mu_{O_{2}}^{\mathrm{at}}$ until equilibrium is reached. Given that the sample consists of two different oxides, each single material thus equilibrates with the atmosphere until $\mu_{O_{2}}^{\mathrm{at}}=\mu_{O_{2}}^{\mathrm{STO}}=\mu_{O_{2}}^{\mathrm{MIEC}}$ .

The chemical potentials of holes and vacancies, μ_h_ and μ_v_, respectively, are linked through the oxygen exchange reaction $\frac{1}{2}O_{2}+v_{O}^{\cdot \cdot }⇌2h^{\cdot }$ , which can be written in terms of chemical potentials as $\frac{1}{2}\mu_{O_{2}}+\mu_{v}=2\mu_{h}$ . In equilibrium with the gas phase, the defect chemical potential differences between STO and the MIEC are thus described by

4 $$\mu_{h}^{\mathrm{MIEC}}-\mu_{h}^{\mathrm{STO}}=\frac{1}{2}\left(\mu_{v}^{\mathrm{MIEC}}-\mu_{v}^{\mathrm{STO}}\right)$$

If STO and the MIEC are in contact, also a defect chemical equilibrium is reached. This, however, requires the formation of a space charge zone and thus establishing of an electrostatic potential difference between bulk STO and the MIEC, as charged species are transferred. Consequently, the charged holes and vacancies have to be described by the electrochemical potential $\mathrm{\mu ̃}=\mu^{0}+kT\ln\left(\frac{c}{1-c}\right)+ze\phi$ , where *c* is the normalized charge carrier concentration and *z* is the charge number of the considered defect. For the holes in equilibrium, i.e., ${\mathrm{\mu ̃}}_{h}^{\mathrm{STO}}={\mathrm{\mu ̃}}_{h}^{\mathrm{MIEC}}$ , the electrostatic potential difference between MIEC and STO thus correlates with $\mu_{h}^{\mathrm{MIEC}}-\mu_{h}^{\mathrm{STO}}$ through

5 $$\mu_{h}^{\mathrm{MIEC}}-\mu_{h}^{\mathrm{STO}}=-e\left(\phi^{\mathrm{MIEC}}-\phi^{\mathrm{STO}}\right)$$

The same considerations for the vacancies lead to the relation

6 $$\frac{1}{2}\left(\mu_{v}^{\mathrm{MIEC}}-\mu_{v}^{\mathrm{STO}}\right)=-e\left(\phi^{\mathrm{MIEC}}-\phi^{\mathrm{STO}}\right)$$

These relations are discussed in more detail in our previous work.^29^ It is essential to emphasize that there is no ″conflict of interest″ between hole and oxygen vacancy equilibration, since eq 5 includes eq 6 due to the validity of eq 4, i.e., equilibration with the gas phase. Thus, the interfacial potential variations can be described either through ionic or electronic charge carriers, i.e., μ_h_ or μ_v_, respectively, both leading to the same result. In the following, the electrostatic potential variation and thus also the STO space charge potential Δϕ will be discussed by focusing on the holes and thus on electronic band alignment.

For the investigated space charge zones, the positive sign of the STO space charge potential (Δϕ > 0) indicates a more positive ϕ in the MIEC. Consequently, μ_h_ is higher in the bulk of STO compared to LSF or LSM. This can be described by a Fermi level E_F_ = −μ_h_ being lower in STO before band bending takes place. From the value of the space charge potential, i.e., the distance between the Fermi levels of STO and MIEC, we can further conclude that E_F_ of solo LSF is lower than E_F_ of solo LSM, as the measured space charge potential for LSM is larger than for LSF. The Fermi levels are thus arranged according to $E_{F}^{\mathrm{STO}}<E_{F}^{\mathrm{LSF}}<E_{F}^{\mathrm{LSM}}$ , as shown in Figure 11a. In this figure the valence band of LSF and LSM is replaced by a narrow energy region representing the hole polaron energies. Defect chemical knowledge also enables us to compare the valence band edges (or polaron levels) $E_{\mathrm{VB}}=-\mu_{h}^{0}$ of the three materials since $\mu_{h}=\mu_{h}^{0}+kT\ln\left(\frac{c_{h}}{1-c_{h}}\right)$ . On the one hand, the concentration of holes in (bulk) LSF and LSM is close to the acceptor dopant concentration in the given *p*(O_2_) range and thus known. On the other hand, from the defect chemical model of nominally undoped SrTiO_3_ we can calculate the hole concentration in STO. Thus, we can suggest the band schemes shown in Figure 11a.

**Figure 11:**
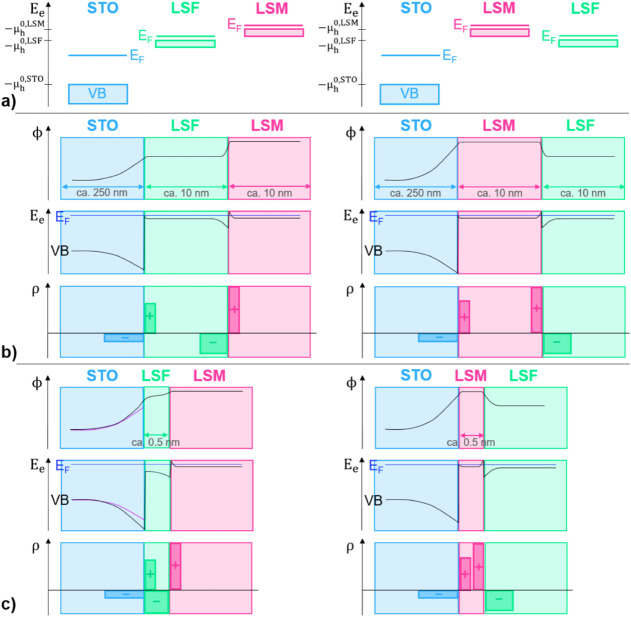
(a) Illustration of the positions of the Fermi levels E_F_ and the according valence band edges $-\mu_{h}^{0}$ (i.e., polaron levels for LSF and LSM) in the sample stacks STO|LSF|LSM and STO|LSM|LSF without defect chemical equilibration (alignment) between the components. b) Illustration of the potential ϕ and the band bending after equilibration, as well as the corresponding charge carrier density ρ indicating the negative and positive excess charge for depletion and accumulation zones, respectively. c) Illustration of ϕ, band bending and ρ for very thin interlayers (≤1 nm). The purple line for STO|LSF|LSM indicate ϕ and band bending in STO for much thicker interlayers (see (b)). Thicknesses are not to scale; for example the space charge layer in STO is much thicker than sketched here. Also the charge densities are not to scale; the individual charges at the STO|MIEC interfaces are much smaller than those at the LSF|LSM interfaces (indicated by the somewhat different area).

Based on this knowledge, we may now discuss the samples with MIEC interlayers, i.e., two MIECs on STO. In contact, the Fermi levels at each interface have to align, confer Figure 11b, leading either to depletion or to accumulation of charge carriers. Due to STO having the lowest E_F_ of the investigated materials, a depletion of holes in the space charge zone is expected in STO for all sample types investigated here and this is what we see as *R*_SC_ and describe by Δϕ. (We included some additional experimental data in the Supporting Information, S3, to show that the influence of surfaces states is of minor importance here.)

### Discussion of the Critical Thickness

Owing to the very high doping concentrations of LSM and LSF, the interfacial ″space charge layers″ in LSF or LSM are drastically thinner than the space charge layer in STO and their resistance is assumed to be negligible in the impedance data. Still they are essential for reaching constant Fermi levels also in the interlayer and top layer. In Figure 11b the corresponding charges and potential steps, as well as the resulting band schemes (valence band edges) are sketched. Owing to the rather different valence band edges (or polaron levels) of LSM and LSF we get a continuous increase of ϕ for STO|LSF|LSM and a maximum of ϕ in LSM for STO|LSM|LSF. It is also important to note that the interlayers always exhibit hole accumulation at the interface to STO (unless space charges begin to overlap, see below). LSF|LSM interfaces, on the other hand, are always characterized by a hole depletion on the LSF side and a hole accumulation in LSM. Owing to the more pronounced charge screening, accumulation layers are thinner than depletion layers, see below. This is often expressed in terms of characteristic lengths. Accumulation is described by the Debye length, which is smaller than the space charge thickness of the Schottky model, eq 2, representing depletion.

For the heterolayers considered here, the measured space charge potential in STO is solely caused by the interlayer, as long as the charges at the two interfaces (STO|MIECI and MIECI|MIECII) are well separated by a sufficiently thick interlayer. This separation of the two interfacial charge zones seems to persist in STO|LSM|LSF down to approximately 0.5 nm LSM, since the space charge potential in STO is almost independent from the LSM film thickness, cf. Figure 10. This is possible, because LSM has the highest E_F_ of the investigated materials (before defect equilibration) and thus accumulation space charge zones form on both interfacial sides of LSM interlayers, as schematically sketched in Figure 11c. In these accumulation space charge zones, the concentration of holes *N*_h_ exceeds the concentration of dopants *N*_d_ and the corresponding space charge thickness can be approximated by the Gouy–Chapman model^42,43^ through the Debye length

7 $$\lambda_{D}=\sqrt{\frac{\varepsilon_{0}\varepsilon_{r}kT}{2e^{2}N_{h,\infty }}}$$

with *N*_h,∞_ being the bulk concentration of the enriched majority charge carrier, i.e., the holes. According to the defect chemical models of LSM^21^ and LSF,^18^ the hole concentration in the bulk corresponds to the dopant concentration of ca. 5.9 × 10^21^ cm^–3^. The resulting accumulation space charge thickness λ_D_ is thus 0.17 nm for an estimated *ε*_r_ of 100. Hence, even LSM layers of 0.5 nm thickness can contain two positively charged accumulation layers (at least in accordance with the continuum model used here). This agrees with our hypothesis that the space charge layers in LSM barely overlap, even at 0.5 nm thickness. Polaronic localization of holes may complicate the situation and impede application of continuum models at this length scale but the main considerations remain valid, see below.

For the LSF interlayer, a change in Δϕ in STO compared to thick LSF films is already noticeable for thicknesses around 1.5 nm. A 0.5 nm LSF interlayer with LSM top layer leads to a Δϕ in STO which is much closer to solo LSM thin films, than to solo LSF thin films. According to the following argumentation this can be attributed to the depletion of holes in LSF at the interface to LSM. The amount of charge *Q* being redistributed at the LSF|LSM interface in order to align the Fermi levels has to be the same in LSF and LSM. For the continuum Mott–Schottky approximation, i.e., a depletion space charge zone, this charge *Q*_S_ per area is given by

8 $$\left|Q_{S}\right|=w_{\mathrm{SC}}eN_{d}$$

with w_SC_ (eq 2) being dependent on the electrostatic potential change Δϕ_S_ in the Schottky-type depletion zone in LSF. The charge for a Gouy–Chapman case *Q*_G_, i.e., for the accumulation space charge zone in LSM, can be calculated from the solution of Poisson’s equation.^42,44^ *Q*_G_ is given by

9 $$\left|Q_{G}\right|=\frac{\varepsilon_{r}\varepsilon_{0}}{\lambda_{D}2k_{B}T}\sinh\frac{\left|\Delta \phi_{G}\right|}{2k_{B}T}$$

where Δϕ_G_ is the potential change across the accumulation zone, e.g., in LSM for LSF|LSM interfaces. The total potential change ϕ^LSM^ – ϕ^LSF^ across the LSF|LSM interface is solely given by $-\left(\mu_{h}^{\mathrm{LSM}}-\mu_{h}^{\mathrm{LSF}}\right)/e$ . This corresponds to the difference of their Δϕ values in STO (cf. eq 5) and amounts to ca. 0.35 V (see Figures 9 and 10). It can be split into the contributions of the depletion and the accumulation space charge zone as ϕ^LSM^ – ϕ^LSF^ = |Δϕ_G_| + |Δϕ_S_|. For the equilibrium condition |*Q*_S_| = |*Q*_G_|, we find a |Δϕ_S_| in LSF of 0.238 V and a |Δϕ_G_| in LSM of 0.112 V. The according space charge thicknesses can be calculated from eqs 2 and 7 and result in 0.66 and 0.17 nm for the depletion zone in LSF and the accumulation zone in LSM, respectively. The corresponding charges |*Q*_S_| = |*Q*_G_| are 3.7 × 10^14^ e/cm^2^.

This means that for very thin LSF interlayers, such as 0.5 nm, the two space charge regions in LSF, i.e., the accumulation space charge at the STO|LSF interface (approximately 0.17 nm) and the depletion space charge zone at the LSF|LSM interface (approximately 0.66 nm) overlap in a continuous model, as shown schematically in Figure 11c. This also affects the STO space charge, since the band alignment at the LSF|LSM interface requires hole depletion in the entire 0.5 nm LSF thin film (more toward the LSM interface but also toward the STO surface). As a consequence, E_F_ of LSF at the STO|LSF interface is higher above E_VB_ than in bulk LSF and thus LSF becomes more ″LSM-like″ when equilibrating defects in contact with STO, cf. Figure 11c. Also the potential difference between LSM and LSF becomes smaller than for thicker interlayers.

The assumption of strong hole depletion in the entire LSF interlayer is also supported by the comparison of the charges resulting at the two interfaces of a thicker LSF film. Above, it was already calculated that LSF has to carry ca. −3.7 × 10^14^ e/cm^2^ as a negative charge from the band alignment with LSM. The space charge potential in STO of 0.55 V corresponds to w_SC_ of 150 nm and thus *Q*_S_ in STO is 6.1 × 10^12^ e/cm^2^, respectively. This charge even includes both hole and oxygen vacancy accumulation. Thus, for a very thin LSF interlayer, the negative charge from the LSF|LSM interface strongly overcompensates the much smaller positive hole accumulation coming from STO and we have a LSF film with very few holes. Fermi level alignment at the LSF|STO interface thus requires a larger space charge potential in STO and this leads to a much larger space charge resistance. For very small ϕ^LSM^ – ϕ^LSF^ we get a Δϕ in STO as for an LSM|STO interface, in accordance with the trend of our measurements.

So far, continuum models were assumed to be applicable to these interfacial space charges. This is certainly the case in our STO single crystals but comes to its limit for the highly doped MIECs. Discrete considerations may thus help to further describe the interfaces of very thin interlayers. We consider holes to be located at either Fe or Mn in LSF or LSM, respectively. This corresponds to a reduction or oxidation of the B cation, i.e., Fe^3+/4+^ or Mn^3+/4+^ in the perovskite lattice, as illustrated in Figure 12. (In the case of Fe, substantial covalency between transition metal and oxygen is present and this is a simplified view.^45,46^ ) For thin films with a thickness of one perovskite unit cell (0.4 nm) this means that there is nominally only one B cation available to carry charges due to band alignment by hole accumulation or depletion. For a one unit cell thin LSF interlayer, see Figure 12a, the Fe cation has to accommodate holes from its accumulation ″space charge″ stemming from the STO|LSF interface. This amount of holes coming from STO is much smaller than the amount of already existing holes in LSF (2.6 × 10^14^ cm^–2^ for 40% Sr in the monolayer). Even the total space charge in STO, including both hole and vacancy depletion is only 6.1 × 10^12^ cm^–2^ or 7.6 × 10^12^ cm^–2^ for 550 mV and 850 mV space charge potential in STO, respectively. However, the LSF monolayer becomes also depleted of holes due to the LSF|LSM interface (ca. 3.7 × 10^14^ cm^–2^ in the continuum model). This depletion requires much more charge compared to the STO-induced accumulation, and thus the LSF unit cell finally exhibits much less holes than bulk LSF. (Please note that the depletion goes even beyond the doping concentration in a monolayer, which is 2.6 × 10^14^ cm^–2^ for 40% Sr, indicating the validity limits of continuous models.) A much lower hole concentration would lead to a much higher Fermi level in LSF, since $E_{F}=-\mu_{h}=-\mu_{h}^{0}-kT\ln\left(\frac{c_{h}}{1-c_{h}}\right)$ . A higher E_F_ in turn requires effectively more space charge in STO and thus a wider space charge zone and a higher space charge potential. This is exactly what is measured experimentally for very thin LSF interlayers.

**Figure 12:**
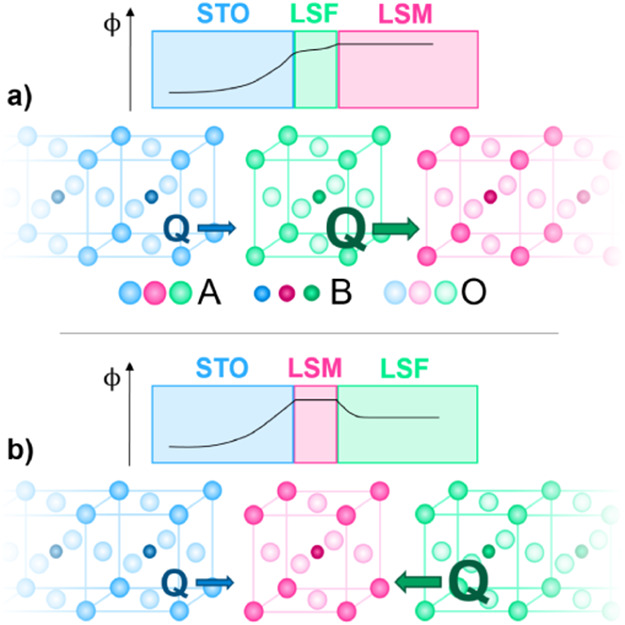
(a) Illustration of the potential ϕ for STO|LSF|LSM with a one unit cell thin LSF, all in contact, as well as an illustration of the perovskite crystal lattice for the investigated heterostructure. Q represents the positive charge, i.e., electron holes. The size of Q and the corresponding arrows symbolize little charge transfer between STO|MIEC (due to low N_d_ in STO) and comparatively strong charge transfer between LSF|LSM. b) Illustration of ϕ for STO|LSM|LSF with unit cell thin LSM in contact and the corresponding illustration of the crystal lattice.

Contrary to LSF, the Mn cation in a very thin LSM layer has to accommodate holes from two accumulation layers, i.e., from the interface with STO (≪ 6 × 10^12^ cm^–2^) and from the interface with LSF (ca. 3.7 × 10^14^ cm^–2^), as sketched in Figure 12b. Because of the already very high doping concentration in LSM (35%, i.e., ca. 5.9 × 10^21^ cm^–3^ and thus 2.3 × 10^14^ cm^–2^ in a unit cell layer), the additional holes from the space charge regions cause only moderate relative concentration changes, which do not have a severe effect on the space charge region in STO. Hence, from both continuous and discrete charge accumulation and depletion considerations we can interpret the different ″critical thicknesses″ of LSF and LSM interlayers. We also believe that this interpretation may play an important role for other material combinations and may help finding and tailoring heterolayers and also surface decorations with properties strongly differing from bulk properties. Still, other factors may also play a role, such as different strain states,^47,48^ confinement-dependent defect concentrations or concentration dependent hole activity coefficients in the MIECs.

Finally, we may also reconsider that fact that even nominal LSM thicknesses of 0.1 nm, i.e., 25% of a monolayer affects the space charge resistance by a factor of 3.5 compared to solo LSF, which corresponds to an effective Δϕ increase of 100 mV. This is assumed to be due to the large space charge thickness of >100 nm (in STO). For LSM islands with distances much below 100 nm those islands affect the entire space charge zone, leading to laterally inhomogeneous potential distributions and an increase of the effectively relevant Δϕ.

## Conclusion

LSM|LSF and LSF|LSM thin film heterostructures were deposited onto STO (100) single crystal substrates by pulsed laser deposition. The interfacial STO space charge regions were investigated by means of impedance spectroscopy at 500 °C in a *p*(O_2_) of 1 to 5 × 10^–4^ bar. Solo LSF layers on STO exhibit a smaller space charge potential than LSM layers, yielding 527 mV compared to 892 mV at 1 bar *p*(O_2_). Heterostructures with 10 nm interlayers still display space charge potentials similar, if not identical, to those of much thicker films. For heterostructures with much thinner interlayers, LSF and LSM behave very differently. LSM interlayers as thin as 0.5 nm, which is close to one unit cell of LSM, still show a space charge effect similar to that of bulk like LSM layers, despite a thick LSF top layer. In contrast, the STO space charge potentials caused by LSF interlayers are affected by LSM top layers already for LSF interlayers of approximately 1.5 nm. A 0.5 nm LSF interlayer beneath an LSM top layer was almost without any effect on the STO space charge.

A model is proposed to describe and interpret the different dependencies of the space charge potential on the interlayer thickness and particularly the different critical thicknesses of LSF and LSM. This model relies on the assumption, that either ionic or electronic charge carriers can be used to describe the space charge potential in equilibrium with the surrounding atmosphere. By focusing on the electronic charge carriers, the alignment of the Fermi levels is used to predict the formation of depletion or accumulation zones at the sample interfaces. Decisive for the different critical thicknesses of LSF and LSM is the fact that at LSF|LSM interfaces hole accumulation takes place in LSM and hole depletion in LSF. The latter requires much thicker space charges and thus interfacial charge layers in LSF begin to overlap already for approximately 1 to 1.5 nm films. Thus, very thin LSF interlayers between LSM and STO become strongly hole depleted, which increases the space charge potential in STO. Calculated and estimated space charge thicknesses indicate the validity of this interpretation.

## Supplementary Material



## Notes

Additional TEM measurements and impedance spectra of a 2.5 nm LSF and 2 nm LSM interlayer sample in dependence of the *p*(O_2_), long-term (>330 h) measurements of LSM interlayer and LSF solo thin film samples, as well as impedance data and the corresponding space charge potentials of MIECs on STO (100) substrates with different terminations (as received and SrO-terminated) (PDF)
