# Supplementary material for: Band Alignment in Ultrathin Mixed Conducting Oxide Layers
Source: ACS Appl Mater Interfaces. 2026 Jun 10;18(24):34596–607. doi: 10.1021/acsami.6c08854 (PMC13307069; doi:10.1021/acsami.6c08854)
Supplement: Supplementary file 1 [file am6c08854_si_001.pdf]

Supporting Information
Band Alignment in Ultrathin Mixed Conducting Oxide Layers

Claudia Steinbach,*,†,‡ Alexander Schmid,† Markus Kubicek,† Andreas Steiger-Thirsfeld,¶ Michael Stöger-Pollach,¶ Alexander K. Opitz,†,‡ and Jürgen Fleig†

†*TU Wien, Institute of Chemical Technologies and Analytics, Vienna, 1060, Austria*

‡*Christian Doppler Laboratory for Oxygen-Ion Batteries, Vienna, 1060, Austria*

¶*TU Wien, University Service Centre for Transmission Electron Microscopy, Vienna, 1040, Austria*

E-mail: claudia.steinbach@tuwien.ac.at

S1. Electron microscopy

Figure S1a shows a high resolution scanning TEM (HRSTEM) image of the interface of the LSF|LSM|STO heterolayer. The surface of the STO single crystal is atomically flat and the mixed conductor layers on top exhibit epitaxial growth. The elemental distribution across the LSF|LSM|STO interface was investigated using electron energy loss spectroscopy (EELS) in parallel to STEM (figure S1, b and c), revealing Mn at very interface. The width (full width at half maximum (FWHM)) estimated through the Gaussian fit, presented in figure S1c, amounts to 1.56 nm. This thickness is even slightly smaller, than expected from quartz microbalance measurements during preparation, which predicted an LSM layer thickness of 2 nm.

The EELS measurement performed across the interfacial region of an LSF|LSM|STO hetero-

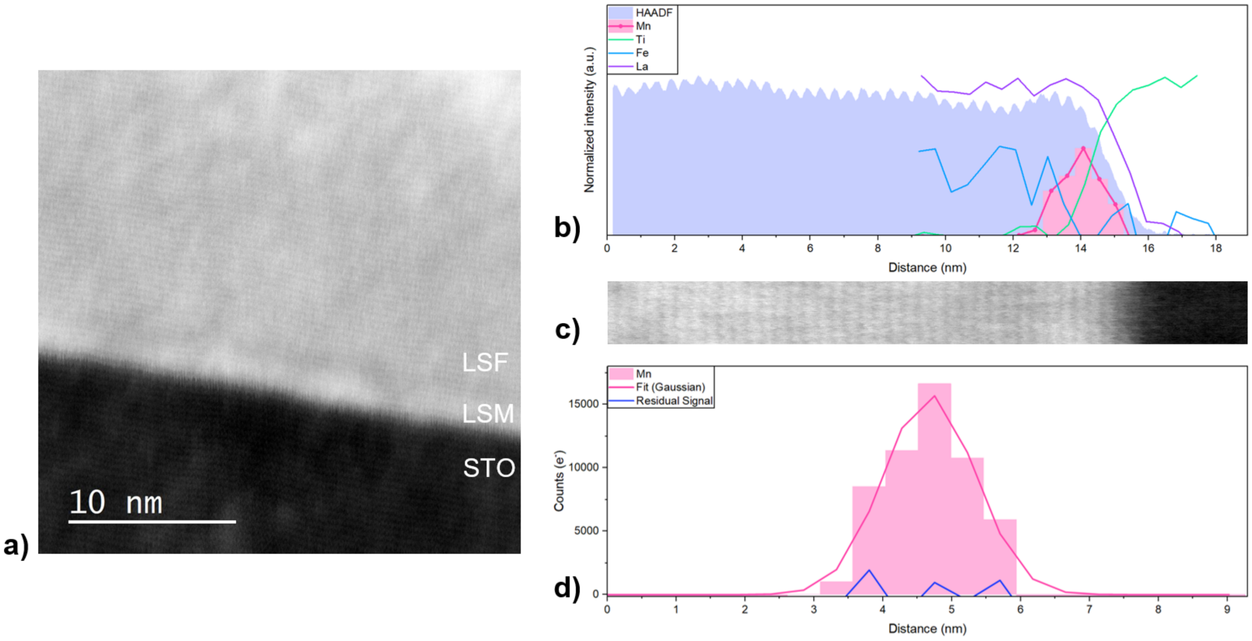

Figure S1: a) High-resolution scanning TEM (HRSTEM) image of the interface of the LSF|LSM|STO heterolayer showing epitaxial growth of the thin films on an atomically flat surface. b) Elemental distribution across the LSF|LSM|STO interface measured with electron energy loss spectroscopy (EELS) parallel to scanning transmission electron microscopy (STEM) revealing Mn at the very interface of the STO single crystal. c) Corresponding STEM detail of b). d) Non-linear least-squares (NLLS) Gaussian fit of the Mn distribution.

layer with a nominal thickness of the LSM interlayer of 0.5 nm (determined by quartz micro balance measurements) is given in figure S2a. The profiles of the ionization edges are enhanced randomly for better visibility. As for the thicker Mn interlayer, Mn is mainly found at the very interface of the sample. The FWHM of the Mn EELS signal is estimated using a Gaussian Fit, shown in figure S2b, amounting to 0.67 nm, which is in excellent agreement with the thickness predicted by the quartz micro balance inside the deposition chamber. This supports our assumption of very little cation interdiffusion at the heterointerfaces.

S2. Impedance in dependence of the $p(O_2)$

The $p(O_2)$ dependence of the interlayer samples is shown exemplarily for the interlayer samples with a 2.5 nm LSF interlayer (LSM on top) and a 2 nm LSM interlayer (LSF on top), both deposited onto polished STO (100) single crystals. Figure S3 shows impedance data taken for the sample

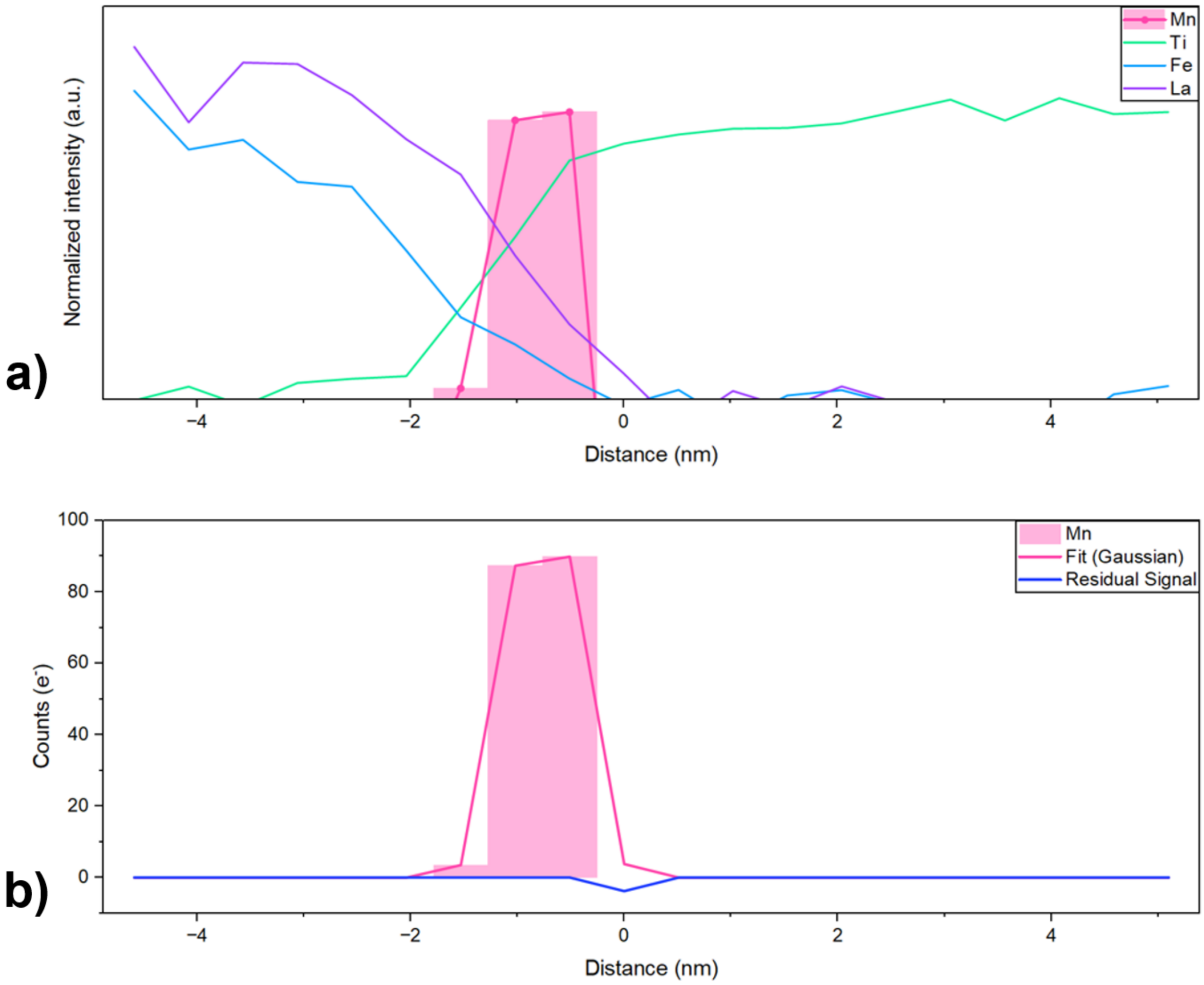

Figure S2: a) EELS measurement across the interfacial region of the LSF|LSM|STO. b) Non-linear least-squares (NLLS) Gaussian fit of the Mn distribution.

with an 2.5 nm LSF interlayer between 1 bar and 0.5 mbar at 500 °C. At 1 bar the smallest resis-

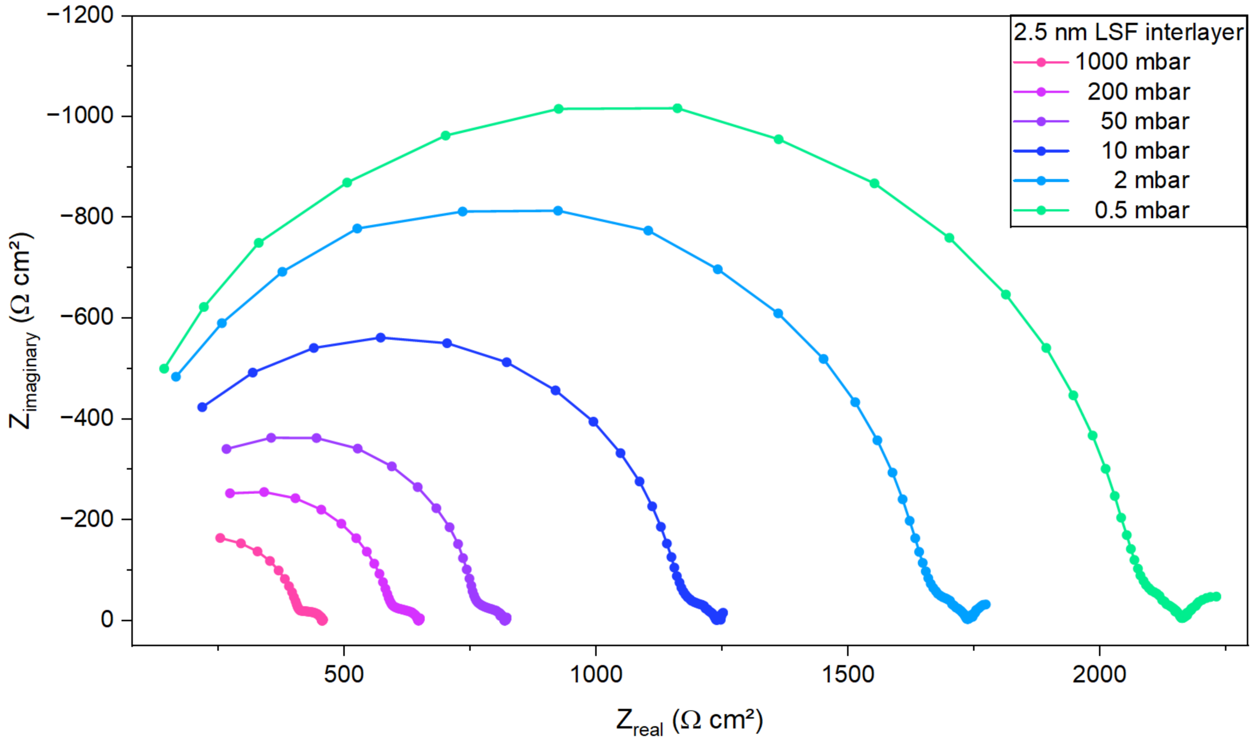

Figure S3: Impedance plots of the LSM|LSF|STO heterolayer with 2.5 nm LSF with LSM on top showing the $p(O_2)$ dependence of the impedance at 500 °C.

tance is measured for both the STO single crystal bulk feature and the space charge feature with 408 Ω cm^2 and 50 Ω cm^2, respectively. With decreasing oxygen partial pressure, the STO bulk resistance increases to 2052 Ω cm^2 and the space charge resistance to 117.5 Ω cm^2, both at 0.5 mbar. The increase of the STO feature is more pronounced compared to the increase of the space charge feature, as also presented in the main part of this publication in figure 7. As a result, the space charge feature merges into the STO bulk semicircle at lower $p(O_2)$ and transforms into a small shoulder of the STO semicircle. At lower $p(O_2)$ a third feature appears in the impedance spectra, which can be assigned to the STO stoichiometry polarization occurring due to blocking of ion transport at the mixed conductor. This is discussed in detail in a previous publication.[1]

Figure S4 shows the $p(O_2)$ dependence of the sample with the 2 nm LSM interlayer and LSF on top, measured at 500 °C. The smallest resistance was again found at 1 bar, yielding 301 Ω cm^2 for the STO single crystal and 1273.5 Ω cm^2 for the space charge feature. Again, the impedance

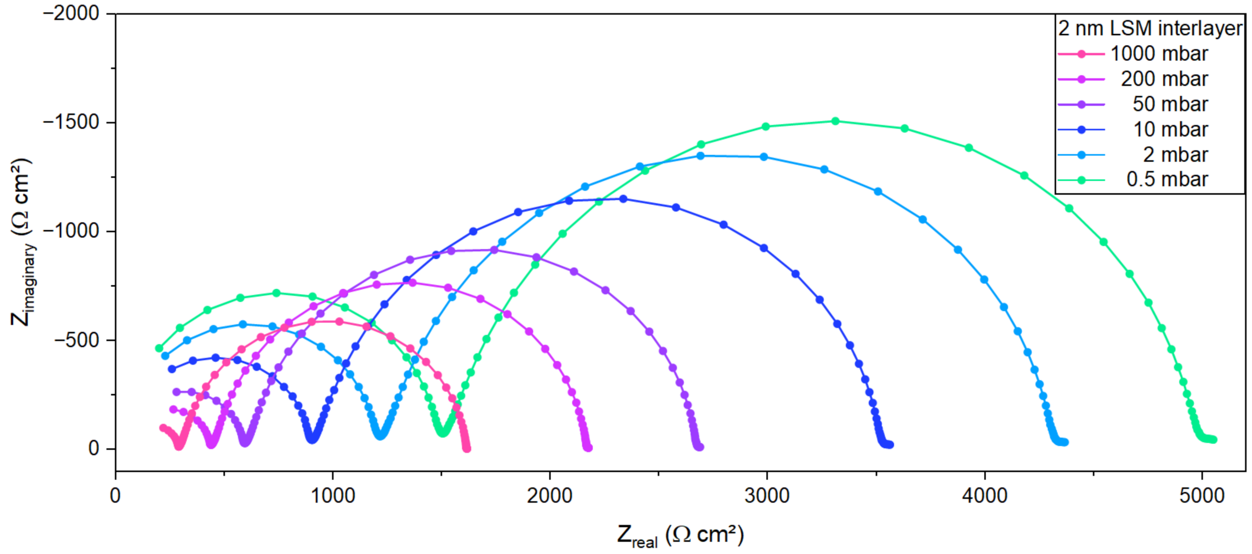

Figure S4: Impedance plots of the LSF|LSM|STO heterolayer with 2 nm LSM with LSF on top showing the $p(O_2)$ dependence of the impedance at 500 °C.

increases towards lower oxygen partial pressure. At 0.5 mbar, the STO bulk resistance amounts to 1531 Ω cm^2 and the space charge resistance to 3306 Ω cm^2. Since the space charge feature shows a large resistance, the third impedance feature occurring due to ion blocking at the mixed conductor can only be recognized as a small extension of the much larger space charge arc at lower $p(O_2)$.

All Nyquist plots were fitted using the nested equivalent circuit described in the main paper, figure 6. Furthermore, as noted in the main part of this publication in the experimental section, the resistance of each sample was tracked over time and a change of less than 1.5 % of the resistance between four consecutive impedance measurements was considered sufficiently equilibrated with the corresponding gas phase.

Figure S5 shows resistance values of the STO single crystal feature R_{STO} and the resistance of the space charge feature R_{SC} extracted from impedance measurements of LSF|LSM|STO interlayer samples with varying LSM interlayer thickness (1, 0.5 and 0.1 nm LSM interlayer, respectively) and an LSF|STO sample without LSM interlayer. Throughout the measurement the temperature was held constant at 500 °C. The samples were investigated in a quadruple impedance measurement setup, allowing all four samples to be in the exact same atmosphere and temperature at exactly the same time, while measuring their impedance consecutively. The experimental pa-

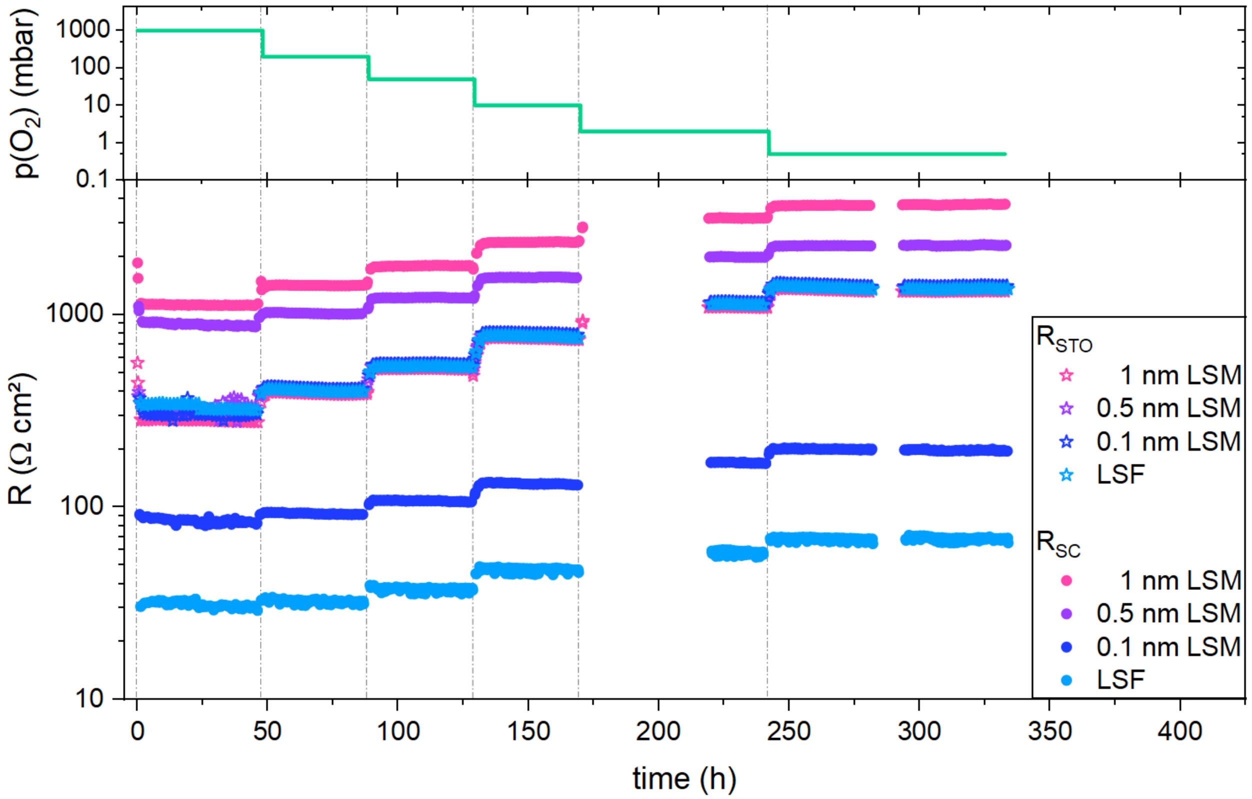

Figure S5: Resistance of the STO single crystal feature R_{STO} and resistance of the space charge feature R_{SC} extracted from impedance measurements of LSF|LSM|STO interlayer samples with varying LSM interlayer thickness and an LSF|STO sample without LSM interlayer. The temperature was held constant at 500 °C, the $p(O_2)$ was varied over time.

rameters of the impedance measurements are given in the experimental section of the main text. During the experiment, the oxygen partial pressure was changed stepwise from 1000 to 0.5 mbar. Please note that the $p(O_2)$ inside the measurement setup was changed using mass flow controllers with a total mass flow of 20 sccm. The measurement setup exhibits a volume of roughly 1 l. Consequently, it can be roughly estimated that establishing of the new oxygen partial pressure inside the measurement setup takes approximately 1 hour.

The increase in the resistance values presented in figure S5 is strongly correlated to the change in $p(O_2)$. After each decrease in $p(O_2)$, both the STO and space charge resistance of the samples exhibit a distinct increase and reach a plateau-like state after roughly 2 to 3 h. When maintaining the oxygen partial pressure constant over several hours, the resistance changes only marginally - if at all. It can therefore be concluded that the major change in resistance can be attributed to changes in partial pressure, and that, on the measured time scale, resistance variations due to possible layer modifications or degradation have only a negligible effect on the resistance and thus the space charge potential. Please note, that at 1000 mbar the visible deviation in the STO resistance between different samples stems from shielding issues of the measurement setup, which were fixed before proceeding to the next $p(O_2)$ (i.e. 200 mbar). The seemingly more prominent scattering of the resistance values of R_{SC} of the LSF sample, compared to R_{SC} for the 1 nm or 0.5 nm, stems from the fact that the space charge semicircle in the impedance measurements of the LSF sample is just a small shoulder of the STO semicircle, which makes fitting more difficult.

S3. Influence of STO substrate changes on the space charge region

In the following, the influence of substrate pretreatment on the space charge region is briefly discussed. Figure S6a shows an AFM image taken for a polished, as received STO (100) single crystal by Crystec (Germany). The root mean square (RMS) roughness is 138.6 pm and terraces of the STO single crystal are detectable. An AFM image of a nominally identical STO (100) single

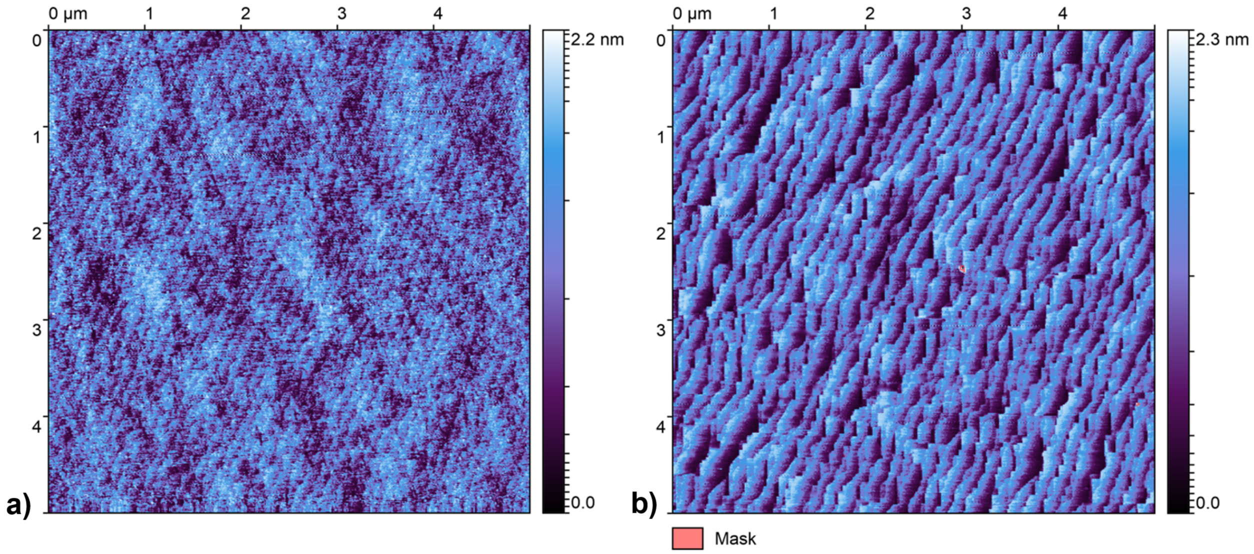

Figure S6: AFM images taken of the surafce of a) an as received STO (100) single crystal by Crystec and b) an STO (100) single crystal that has been pretreated. The masked region in b) was excluded for the determination of the surface roughness.

crystal with pretreatment is given in figure S6b. For the pretreatment, the as received single crystal was cleaned in an ultrasonic bath in 30 % HNO_3 for 30 min, followed by a 30 min ultrasonic cleaning step in ethanol at 60 °C. Subsequently, the single crystal was sonicated two times for 30 min at 60 °C in a 3 % extran solution. To remove any extran residue, the single crystal was then sonicated in bi-distilled water for 15 min. Finally, the single crystal was annealed in a furnace at 1000 °C in air for 8 h. The temperature ramp of the furnace was set to 5 °C/min. Compared to the surface of the as received STO (100) single crystal, the terraces of the pretreated STO are larger and more defined. The RMS roughness yields 172.9 pm. Similar STO AFM images and surface treatments were also described in literature,[2] where comparable terraces were found to be predominantly SrO-terminated. For this reason, the pretreated STO (100) single crystals will be referred to as SrO-terminated in the following. (Please note, however, that additional analytical measurements to confirm a SrO terminated surface were not performed.)

Figure S7 shows exemplarily impedance spectra taken at 1000 mbar and 500 °C for a 50 nm LSF thin film on top of two different, polished STO substrates: on a STO (100) single crystal (Crystec) as received and on a pretreated one. The high frequency arc is the STO single crystal feature. For

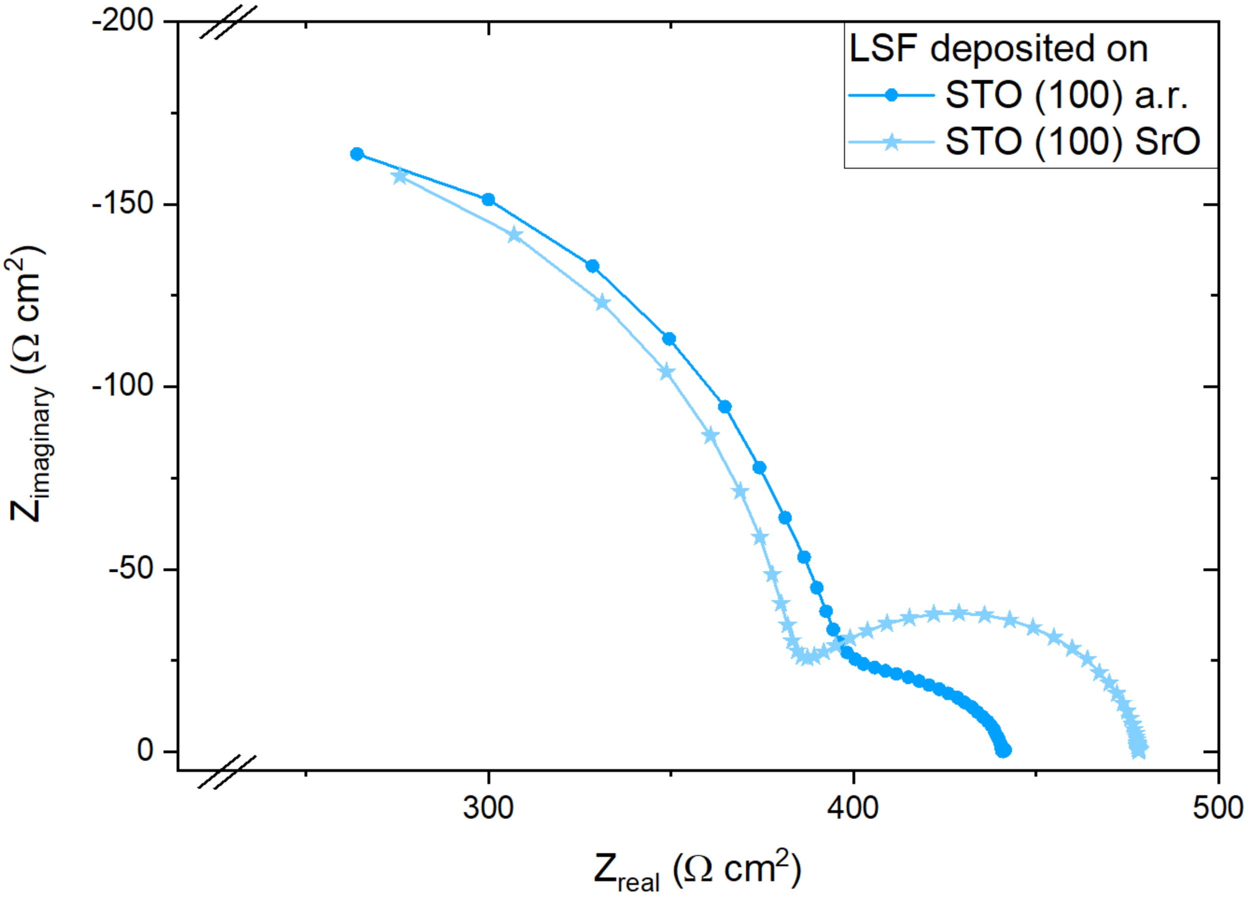

Figure S7: Impedance plots of an LSF thin film (50 nm) on top of two different, polished STO substrates: as received by the manufacturer STO (100) ("a.r.") and pretreated STO (100) ("SrO") at 1000 mbar and 500 °C.

both STO (100) samples, this high frequency feature is very similar in size. The mid-frequency space charge arc exhibits a somewhat larger variation in size, compared to the STO bulk features. The smaller space charge arc is found for the STO (100) as received single crystal with 59 Ω cm^2, while the STO (100) SrO-terminated sample leads to a space charge arc with 105 Ω cm^2. (All impedance spectra have been fitted with the nested equivalent circuit shown and discussed in the main part of this publication.)

The corresponding space charge potentials of the LSF samples (deduced as described in the main part of this publication) are plotted in figure S8. Moreover, space charge potentials deduced

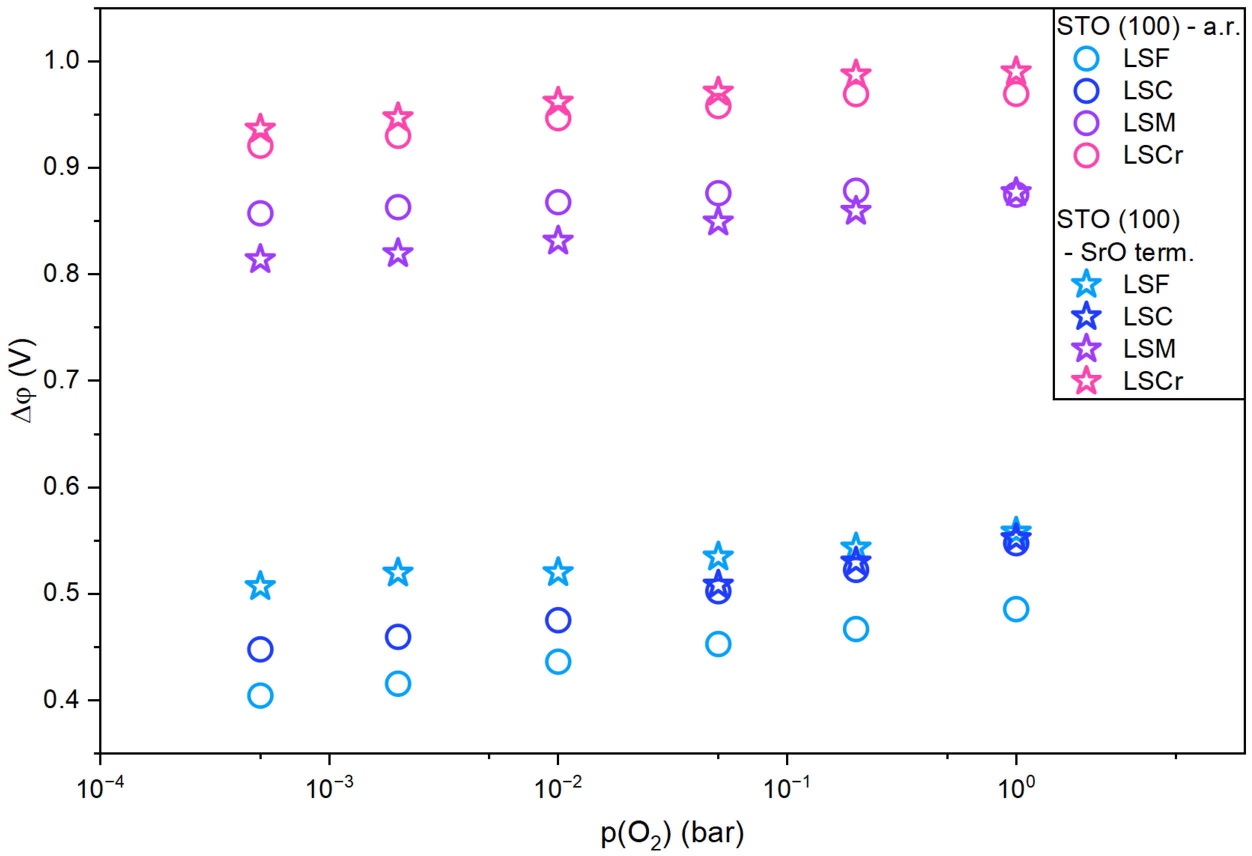

Figure S8: Space charge potentials deduced from impedance measurements taken for LSF, LSC, LSM and LSCr thin films (all ca. 50 nm) on different STO substrates at 500 °C. As received by the manufacturer single crystal substrates are labeled "a.r.". Pretreated STO single crystals are labeled "SrO term.".

from impedance measurements of LSC ($La_{0.6}Sr_{0.4}CoO_3$), LSM and LSCr ($La_{0.9}Sr_{0.1}CrO_3$) thin films (all ca. 50 nm) on STO substrates with different terminations are also given in figure S8. Parameters for thin film depositions are given in a previous work.[3] For the measured LSF samples, the

space charge potentials amount to 0.486 V for the as received STO (100) substrate and 0.558 V on SrO-terminated STO, both at 1000 mbar. For all other investigated thin films on differently pretreated substrates, the difference in the space charge potential is even smaller than for LSF. While LSC on as received STO (100) exhibits a space charge potential of 0.548 V, the space charge potential on SrO-terminated STO was found to be 0.552 V, both at 1000 mbar. For LSCr, the space charge potential for as received STO (100) substrates is also slightly lower than for SrO-terminated STO, amounting to 0.969 V and 0.990 V at 1000 mbar, respectively. For LSM, the largest difference occurred at 0.5 mbar, with a space charge potential for as received STO of 0.875 V compared to 0.814 V for pretreated STO.

With the exception of LSF, the substrate pretreatment does not show a substantial effect on the space charge potential between STO and other MIECs. In our previous publications,[3] it was discussed that deviations between nominally identical samples are around 20 mV. This is roughly in the same order of magnitude as the difference found for most of our STO pretreatments. This suggests that the space charge in STO is indeed mainly determined by the bulk Fermi-levels of the two oxides rather than by interfacial states. Consequently, also in our model considerations we neglect the influence of surface states. However, some interfacial effects may be present in LSF. There, also the effect of the surface orientation was tested and revealed a moderate but measureable difference: space charge potentials of 0.611 V (at 1000 mbar) for as received STO (111) are found.

References

(1) Steinbach, C.; Schmid, A.; Huber, T. M.; Fleig, J. The Oxygen Partial Pressure Dependence of Space Charges at $SrTiO_3$|Mixed Ionic Electronic Conducting Oxide Heterojunctions. *Small Methods* **2025**, e2500728.

(2) Bachelet, R.; Sánchez, F.; Palomares, F.; Ocal, C.; Fontcuberta, J. Atomically Flat SrO-terminated $SrTiO_3$ (001) Substrate. *Applied Physics Letters* **2009**, *95*.

(3) Steinbach, C.; Schmid, A.; Siebenhofer, M.; Nenning, A.; Rameshan, C.; Kubicek, M.; Fleig, J. Space Charges at $SrTiO_3$|Mixed Ionic and Electronic Conducting Oxide Heterojunctions and Their Relation to Defect Chemistry. *ACS Applied Materials & Interfaces* **2025**, *17*, 17543–17557.
